# A Pleistocene Fight Club revealed by the palaeobiological study of the *Dama*-like deer record from Pantalla (Italy)

**DOI:** 10.1038/s41598-022-18091-1

**Published:** 2022-08-16

**Authors:** Marco Cherin, Marzia Breda, Bruno Esattore, Vlastimil Hart, Jiří Turek, Francesco Porciello, Giovanni Angeli, Sofia Holpin, Dawid A. Iurino

**Affiliations:** 1 Department of Physics and Geology, University of Perugia, 06123 Perugia, Italy; 2 University Museum Centre (CAM), University of Padova, 35121 Padova, Italy; 3 Department of Ethology, Institute of Animal Science, 104 00 Prague, Czech Republic; 4 Department of Game Management and Wildlife Biology, Faculty of Forestry and Wood Sciences, Czech University of Life Sciences, 165 00 Prague, Czech Republic; 5 Department of Veterinary Medicine, University of Perugia, 06126 Perugia, Italy; 6 School of Geosciences, University of Edinburgh, Edinburgh, EH9 3FE UK; 7 PaleoFactory, Sapienza University of Rome, 00185 Rome, Italy

**Keywords:** Palaeontology, Palaeoecology

## Abstract

Here, we report on the exceptionally well-preserved deer record from the locality of Pantalla (central Italy), dated in the Early Pleistocene (ca. 2.1–2.0 million years ago). The fossils show a combination of characters that allows an unambiguous attribution to *‘Pseudodama’ nestii*, of which they represent one of the most informative collections to date. Our comparisons—also conducted through CT-based methods on endocranial structures—reveal that the cranial and postcranial skeleton of *‘P.’ nestii* displays a mosaic of intermediate characters between extant *Dama* and *Cervus*, but also that the affinities with *Dama* are prevalent. Some *Cervus*-like features especially in cranial morphology, can be interpreted as plesiomorphic characters supporting a basal position of *‘Pseudodama’* among the Cervini. Interestingly, three bone anomalies are described in the two male crania of *‘P.’ nestii* from Pantalla and are interpreted as palaeotraumatological evidence resulting from different injuries suffered by the deer during their life. This allows opening a treasure trove of information on paleobiological aspects, including ontogeny and antler cycle and function.

## Introduction

Based on molecular data^1,2^, the divergence of *Dama* from *Cervus* can be referred to ca. 5.1 Ma, that is during the early Pliocene, while the divergence between the extant *Dama dama* and *Dama mesopotamica* is estimated to around the middle Pliocene, ca. 4 Ma. However, the earliest fossil remains of *D. mesopotamica* are dated only to the Late Pleistocene^3^ and those of *D. dama* to the late Middle Pleistocene (MIS 11), if we consider *Dama clactoniana* as a subspecies of *D. dama*, but to MIS 7, if we consider *D. d. tiberina* and *D. d. geiselana* as valid subspecies, or even to MIS 5, if we just consider the first occurrence of modern *D. dama*^4^.

Historically, the first mention of the genus *Dama* is that of Frisch^5^, who associated the name to deer having slight or pronounced distal palmation of the antlers. The arbitrary term “*Dama*-like” deer has been used to collectively refer to Plio-Pleistocene cervids roughly the same size of the extant fallow deer, but with three- or four-pointed and un-palmated antlers.

During the Villafranchian European Land Mammal Age (ca. 3.3–1.2 Ma), Epivillafranchian stage (ca. 1.2–0.8 Ma), and early Middle Pleistocene, several *Dama*-like deer species occurred in Europe, but there is still much debate on their taxonomy and relationships. Azzaroli^6^ introduced the genus *Pseudodama* to accommodate all Villafranchian *Dama*-like deer, previously largely referred to as *Cervus*^7–9^ as usual practice up to the early twentieth century. Azzaroli^6^ proposed that after splitting from a common ancestor, *Pseudodama* evolved along two anagenetic lineages: the “Italian” lineage with *P. lyra*, *P. nestii* (type species), and *P. farnetensis*, and the “French” lineage (which follows Heintz’s interpretation^9^) with *P. pardinensis*, *P. rhenana* (= *P. philisi*), and *P. perolensis*. Unfortunately, Azzaroli^6^ did not provide clear comparisons between the Italian and French *Pseudodama*. However, the antlers of both Italian and French species display a gradual modification of the basal tine from the late Pliocene to the Middle Pleistocene, which in earlier forms is placed at a certain distance from the burr and forms an acute angle with the beam, whereas in later forms it gets closer to the burr and forms an obtuse angle with the beam^6,10^. Some of the richest populations include a minor number of specimens with an intermediate morphology or even with a morphology “opposite” to the main one (in terms of opening of the angle and/or its distance from the burr). Examples are *P. nestii* from Upper Valdarno (Italy), where the specimens with “advanced” morphology were initially attributed by Azzaroli^8^ to the subspecies *Dama nestii eurygonos* to be later considered just a morphotype within *P. nestii* pertaining to more aged individuals^11^, and *P. vallonnetensis* from Untermassfeld (Germany), where the specimens with “primitive” morphology were attributed by Croitor^12^ to the species *Metacervoceros rhenanus* (= *P. rhenana*).

The genus *Pseudodama* is used by several authors (e.g.,^13–17^), but the literature has provided several other systematic interpretations over the past 20 years. Some authors preferred to use the name *Dama* for all these species due to similarities with modern fallow deer (e.g.,^10,18,19^), others even assigned the Italian and French lineages to the Asian genera *Axis* and *Rusa*, respectively^20^. Croitor attributed European *Dama*-like deer to a variety of different, apparently unrelated genera (*Cervus*, *Dama*, *Metacervocerus*, *Praeelaphus*), sometimes co-occurring in the same sites^12,21–23^. A similar taxonomic interpretation is also followed in a recent phylogenetic study^24^, in which although the proximity between Plio-Pleistocene *Dama*-like deer and the extant *Dama* and *Cervus* is recognized, the relationships between all these forms are far from resolved.

Since all the species assigned by Azzaroli^6^ to *Pseudodama* share among them and with modern *Dama* many morphological features of the skull, teeth, and postcranials, suggesting they are strictly related to each other (e.g., ^10,25^), we choose here to keep them united in the genus *‘Pseudodama’* instead of splitting them in an array of genera. In addition, we believe that the gradual shifting of the position of the basal tine and its gradual opening is not enough to part the group in an earlier and a later genus but, rather, suggests that specimens of different age are part of the same lineage. This choice accounts for the fact that antlers are plastic structures and although an easier tool to identify species, the uniformity of cranial and postcranial characters between earlier and later forms, should be a priority factor in taxonomical issues. Pending a comprehensive phylogenetic analysis, here we use the name *‘Pseudodama’* within inverted commas following the most recent literature^4,26–29^, to account for the fact that, if modern *Dama* derives from *Pseudodama,* the latter is a paraphyletic stem group. Thus, we consider the following species to belong to this genus (in chronological order): *‘P.’ pardinensis* (Early Villafranchian); *‘P.’ lyra* (Early-Middle Villafranchian); *‘P.’ rhenana* (Middle-early Late Villafranchian); *‘P.’ nestii* (early Late Villafranchian); *‘P.’ farnetensis* (late Late Villafranchian); *‘P.’ perolensis* (late Late Villafranchian); *‘P.’ vallonnetensis* (Epivillafranchian-earliest Middle Pleistocene). A discussion of the possible synonymies of some of these species goes beyond the scope of the present report. In spite of the numerous records of these forms from a number of European sites (Supplementary Table S1), very few works have so far been devoted to palaeobiological aspects^12,28,30–33^.

In this paper, we report on the outstanding cervid record from the Early Pleistocene locality of Pantalla (central Italy) also by CT-based comparison with extant taxa (*Dama* and *Cervus*) and we discuss some palaeobiological issues, including palaeopathology, ontogeny, antler development and function.

## Results

### Systematic palaeontology

- Order Artiodactyla Owen, 1848
- Family Cervidae Goldfuss, 1820
- Subfamily Cervinae Goldfuss, 1820
- Tribe Cervini Goldfuss, 1820
- Genus *‘Pseudodama’* Azzaroli, 1992
- *‘Pseudodama’* nestii (Azzaroli, 1947).

### Description

The cervid sample from Pantalla consists of 22 specimens listed in Supplementary Table S2. The majority of the specimens come from a small (ca. 2 m^2^) bone accumulation within a silty-sand yellowish layer^34^. Sedimentological and taphonomic evidence suggests that this accumulation formed in very short time^34,35^.

**Figure 1:**
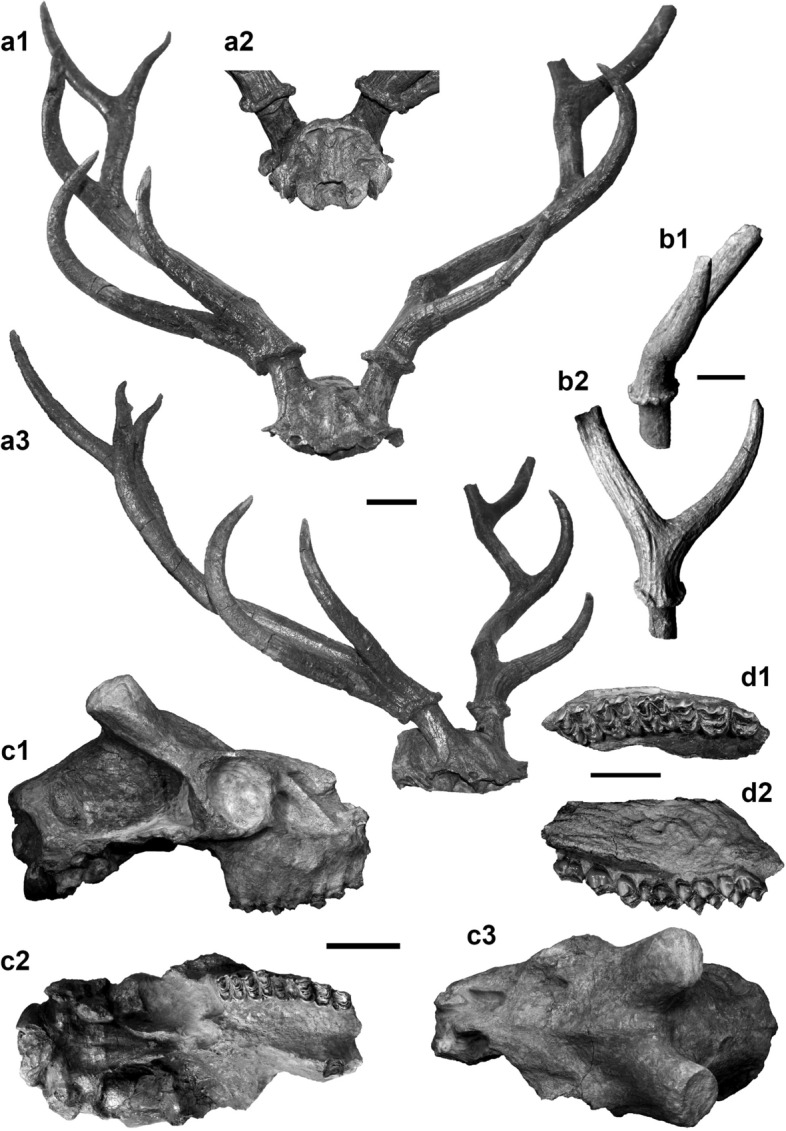
Selected cranial remains of *‘Pseudodama’ nestii* from Pantalla (Italy). (**a**) Cranium SABAP_UMB 337655 in rostral (**a1**), caudal (**a2**), and rostro-lateral (**a3**) views. (**b**) Left pedicle with basal fragment of antler SABAP_UMB 337625 in rostral (**b1**) and medial (**b2**) views. (**c**) Cranium SABAP_UMB 337643 in right lateral (**c1**), ventral (**c2**), and dorsal (**c2**) views. (**d**) Maxillary fragment SABAP_UMB 337633 in occlusal (**d1**) and lingual (**d2**) views. Scale bars: 3 cm.

The sample is made up of 73% cranial remains (Supplementary Table S2) in an exceptional state of preservation. Among these, two adult male partial crania stand out, one with antlers, SABAP_UMB 337655 (the abbreviation SABAP_UMB is omitted hereinafter) and one without antlers, 337643 (Fig. 1a,b). The latter has undergone a plastic laterolateral compression that most probably led to the deformation of some bones (e.g., the narrowing of the palate). In lateral view, 337643 shows a rather long, cylindrical brain case. The pedicles are oval in section and are inclined caudally almost in parallel to the forehead profile. See Supplementary Note 1 for a detailed anatomical description of the specimen.

**Figure 2:**
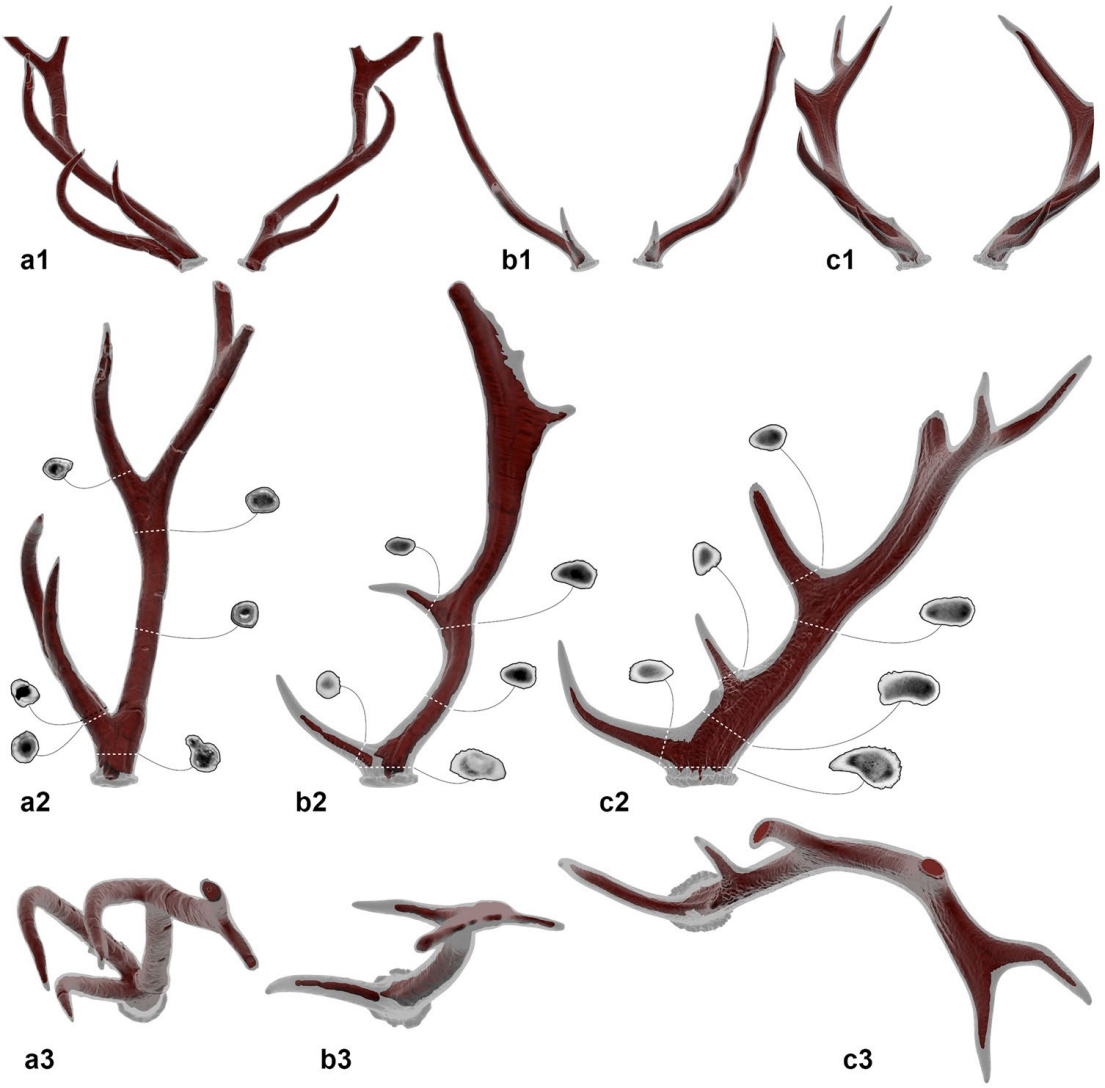
CT-based comparison of antler shape and cross-sections. (**a**) *‘Pseudodama’ nestii* SABAP_UMB 337655; (**b**) *Dama dama* CZUFLD FaD002; (**c**) *Cervus elaphus* CZUFLD ReD001. Antler pairs in anterior (**a1–c1**) view; right antler in medial (**a2–c2**) and apical (**a3–c3**) views. The internal structure of the antlers (spongious regions plus medullary cavities) is highlighted in dark red and through sections. All the images are normalized.

On the male neurocranium 337655 both antlers are preserved, but the entire splanchnocranium is missing (Fig. 1a). The overall state of preservation is very good except for a slight diagenetic right lateral torsion. When compared with 337643, 337655 shows the following differences: in lateral view, the braincase and temporal fossa are shorter; the interfrontal crest on the forehead is lower; in dorsal view, the supraorbital foramina are wider; the distance between pedicles is wider and they have a sub-circular section; the squared prominence in the nuchal interparietal area is much more prominent; in caudal view, the occipital squama is relatively wider and lower; the vertical median bulge of the occipital squama is less prominent, as is the external occipital protuberance. The antlers of 337655 are almost complete (Figs. 1 and 2). They have four tines: a basal tine, a middle tine, and a two-pointed terminal fork. The basal tine is positioned at ca. 3 cm from the burr and branches at an angle of ca. 45° obliquely to the median plane. The middle tine is just over half way up the beam and diverges laterally; it curves medially forming a C-shape and has roughly the same size as the basal tine (see measurements in Supplementary Table S3). The terminal fork is perpendicular to the median plane, rotated almost 90° compared to the basal bifurcation. In rostral view, the beams diverge slightly in the basal portion and more markedly above the basal tine. Above the middle tine, they are subparallel. In lateral view, only the beam is slightly caudally inclined. The sections of the beams and tines are sub-circular in almost their entire length, except near the bifurcations where they flatten slightly (Fig. 2a). Longitudinal grooves run along the entire antler, but are particularly deepened in the basal part. Specimen 337625 (Fig. 1b), a left pedicle and attached basal portion of an antler including a complete basal tine, matches the morphology described for 337655. The latter is the most complete ‘*P.*’ *nestii* specimen recovered so far. The famous type specimen from Upper Valdarno (IGF 363) is in fact less complete in its cranial portions, and its antlers are heavily reconstructed (compare Fig. 4.4 pre-reconstruction in Azzaroli^8^ against Fig. 5 post-reconstruction in Azzaroli^6^).

Detailed descriptions of the upper and lower teeth and of the postcranial material are available in Supplementary Note 1. Selected material is shown in Supplementary Figs. S1 and S2, whereas measurements of all specimens are in Supplementary Table S3. Estimates of age of death based on lower tooth eruption and wear are in Supplementary Table S4.

### Palaeoneurology

**Figure 3:**
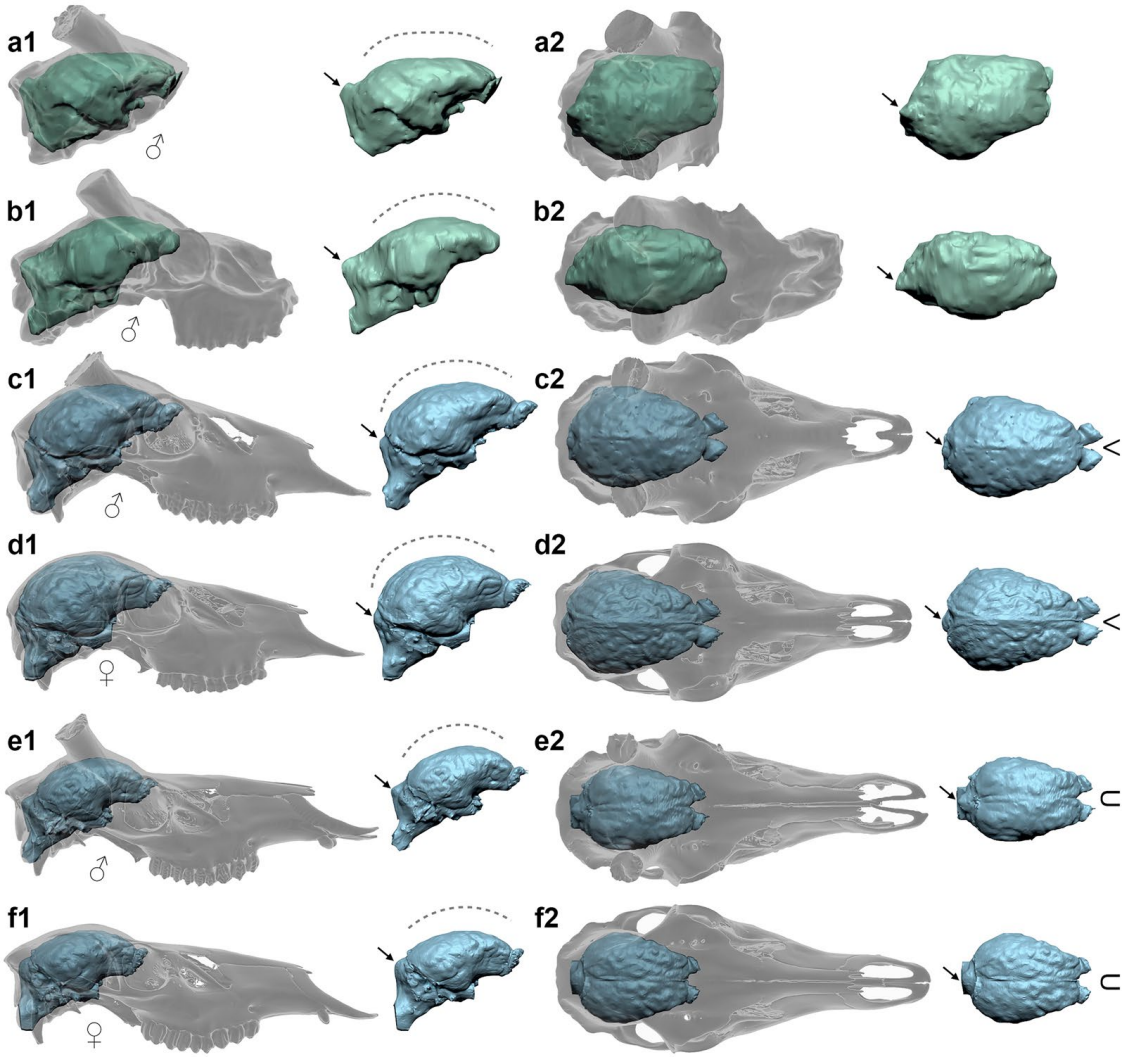
CT-based comparison of crania and digital brain endocasts. (**a**) *‘Pseudodama’ nestii* SABAP_UMB 337643; (**b**) SABAP_UMB 337655; (**c,d**) extant *Dama dama*, male CZUFLD FaD002 (**c**) and female CZUFLD FaD003 (**d**); (**e,f**) extant *Cervus elaphus*, male CZUFLD ReD002 (**e**) and female CZUFLD ReD003 (**f**). Crania and digital models in right lateral (**a1–f1**) and dorsal (**a2–f2**) views. The black arrows indicate the prominence of the cerebellum, while symbols on the right indicate the V or U shape of the olfactory bulbs. All the images are normalized.

Virtual endocasts obtained from 337643 and 337655 present two nearly complete brains, similar in size, but without olfactory bulbs (Fig. 3, Supplementary Table S5). The poor detail of the digital models due to preservation issues and presence of infilling sediment, allowed the identification of the main brain areas but not convolutions. Both brains are anteroposteriorly longish and laterolaterally narrow, consisting of a dorsally flattened telencephalon, a prominent and well-developed cerebellum, and a portion of the brain stem. Only few brain endocasts of fossil Cervidae are known from the Plio-Pleistocene of Europe. Of these, a perfectly preserved natural endocast (IGF 2175) from Castelfiorentino (Italy) was comprehensively described by Beccari^36^, who provided one of the first and most informative contributions in ungulate palaeoneurology of that time. The large size of IGF 2175, its strong dorsoventral slenderness, and the peculiar pattern of convolutions*,* prompted the author to tentatively ascribe this sample to *“Cervus” dicranius* (= *Eucladoceros dicranius*). Compared with the endocasts from Pantalla this brain is considerably larger (Supplementary Table S5) and slenderer. After Beccari’s work, two partial natural brain endocasts (IGF 1419, IGF 1422) of *‘P.’ nestii* from the Early Pleistocene of Olivola (Italy) were described by Azzaroli^8^ and reviewed by the same author^6^ with updated figures and some comments on the Castelfiorentino specimen. Only the endocast IGF 1422 is sufficiently preserved to deliver measurements and to appreciate its overall aspect, including the main convolutions. The cerebrum from Olivola reaches the size of that of extant male *Cervus elaphus* (especially in length), and is slightly larger than those from Pantalla (Supplementary Table S5). However, regardless of size, in both the Pantalla and Olivola samples the telencephalon is dorsoventrally flattened and rostrally longish. The cerebellum is incomplete in IGF 1422 and completely missing in IGF 1419.

Comparisons with extant specimens of *D. dama* and *C. elaphus* by CT scans (Fig. 3c–f), show some morphological similarities between the Pantalla sample and the brain of the red deer, such as the longish shape of the cerebrum (which however in the extant species never reaches the dorsal flattening observed in *‘P.’ nestii*) and a prominent cerebellum with a marked vermis. In contrast, *Dama* has a more rounded cerebrum with a compact and less projected cerebellum (Fig. 3c,d). A certain dorsoventral flattening of the cerebrum is also showed by *“Cervus” warthae* (= *Praeelaphus warthae*^17^) from the Pliocene-Early Pleistocene of Węże-1 (Poland)^37^, in which however, the cerebellum is not preserved. In terms of volume and proportions (i.e., ratio between the breadth and length of the telencephalon), the endocasts of Pantalla match the values of extant *D. dama* (Supplementary Table S5). Moreover, in extant species the margin between the olfactory bulbs in dorsal view is V-shaped in *Dama* and U-shaped in *Cervus,* and in both the brain size is slightly larger in males than in females.

### Palaeotraumatological evidence and antler anomalies

**Figure 4:**
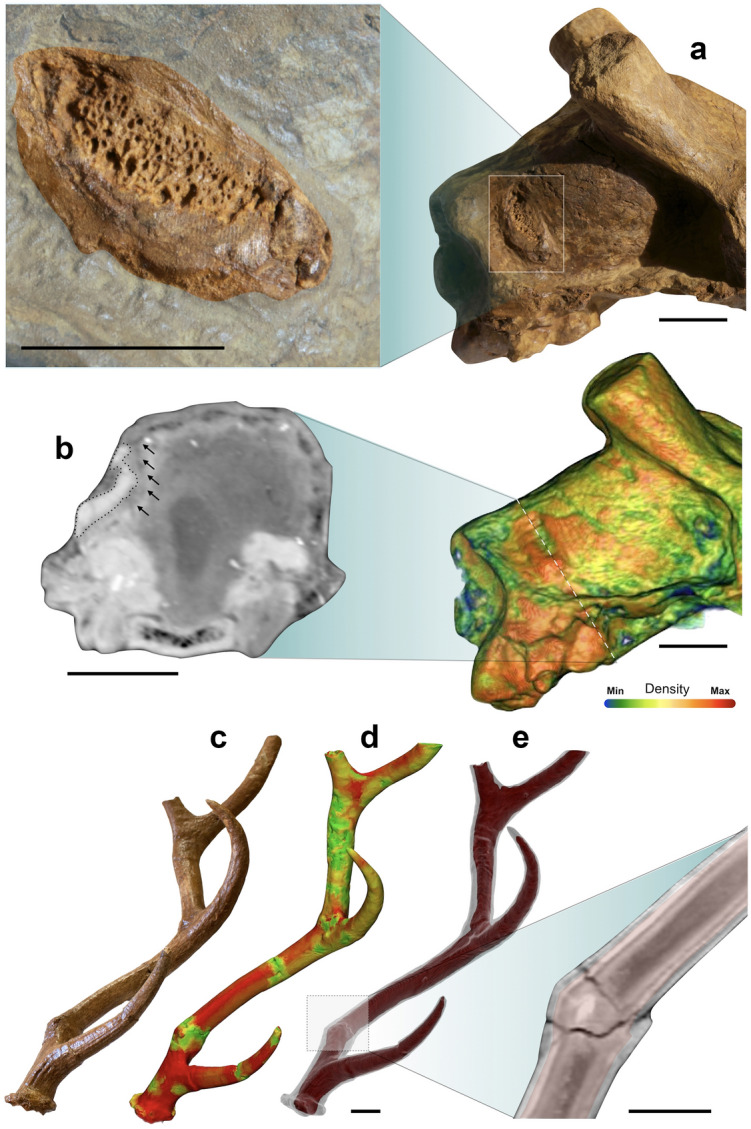
Palaeotraumatological evidence of the Pantalla specimens. (**a**) Traumatic injury with magnification of the bone callus on SABAP_UMB 337643. (**b**) Bone density filter applied on the same specimen with magnification of the cranial cross-section (dotted line) at the level of the lesion; black arrows indicate the depression on the left hemisphere. (**c–e**) Injured left antler of SABAP_UMB 337655 (**c**), with density filter (**d**) and magnification of the fracture and bone callus (**e**). Scale bars: 3 cm.

The male cranium 337643 shows a bone anomaly caudodorsally to the right squamosal, close to the nuchal crest (Fig. 4a). It appears as a dorsoventrally arranged ovoid-shaped structure, ca. 3 cm in maximum length, delimited by a clear-cut line of laminar bone, with a slight prominence caudally. Spongious bone was accidentally exposed in its centre during preparation. CT images (Fig. 4a,b) show that the prominence and bone tissue underlying the spongious area are made of very dense bone interpreted as a callus that caused deformation of the brain cavity wall. This deformation is recorded on the 3D brain endocast, appearing as a slight depression on the caudal portion of the right hemisphere (Figs. 3a, 4b). The shape of the callus and its position are consistent with a traumatic injury inflicted by a sharp object as the tip of an antler.

The cranium 337655 is notable not only for the extraordinary preservation of the antlers, but also for their atypical morphology. The left antler beam of 337655 shows a swelling and a kink ca. 5 cm above the first bifurcation, corresponding to an anomalous change in orientation of the beam downwards (Fig. 4c–e). The inner structure of the swelling shows an incomplete fracture surrounded by a thick matrix of newly formed bone tissue (Fig. 4c–e) indicating an *ante mortem* origin, hence a trauma. There are several other fractures on both antlers, some directly visible on the surface of the sample, others only through tomographic images, but all of taphonomic origin as confirmed by the lack of reactive bone tissue. The right antler of the same specimen presents a supernumerary tine (Figs. 1a, 2a). The base of the beam has a thick section and the rim of the burr appears indented. The aberrant supernumerary tine splits lateral from the basal bifurcation. It is longer and more curved with respect to all the other tines. Basally it is almost horizontally arranged, then it curves apically and then slightly medially, the tip reaching back almost a third of the total length of the antler. In apical view, the beam, basal tine, and extra-tine form a trifurcation (Fig. 2a). The inner structure of the extra-tine (Fig. 2a) is comparable to that of the rest of the antler, though a more massive development can be pointed out.

## Discussion

### Taxonomy, variation, and biochronology

The fossils described herein represent one of the most valuable and best-preserved samples of “*Dama*-like” deer from the European Early Pleistocene. The systematics of these forms has been essentially based on the morphology of the antlers and teeth, with less attention paid to the skull (due to the rarity of well-preserved finds) and postcranial bones.

The Pantalla sample shows a combination of characters allowing an unambiguous attribution to ‘*P.’ nestii*, a species reported confidently so far in the early Late Villafranchian of Italy (several sites) and in the Georgian *Homo*-bearing locality of Dmanisi (Supplementary Table S1). Based on the literature^6,8,12,38^, these characters include: four-pointed antler with elongated, slender, and tubular beam; basal tine branching off at a certain distance from the burr forming an acute angle; well-developed middle tine; terminal bifurcation oriented normal to the sagittal plane; cranium with large orbits, preorbital fossae, and ethmoidal vacuities; relatively elongated neurocranium with flat parietals; caudally-oriented pedicles; molarized P2-P3; presence of cingula in upper molars; enlarged i1; un-molarized p4. However, some characters observed in the Pantalla specimens (e.g., rostral edge of the orbit reaching the level of M2; elongated metapodials) do not fit the revised diagnosis of ‘*P.’ nestii* by Croitor^12^. The latter author considers *nestii* as the earliest species of the genus *Cervus* based on similarities with the extant red deer especially in cranial morphology^12,22,23^. However, in our opinion, his conclusions are biased by relying mostly on the skull IGF 243 of ‘*P.’ nestii* from Upper Valdarno^6,8^, which is heavily deformed and belongs to a juvenile individual (see below for details on ontogenetic variation in *‘Pseudodama’*).

A broader look at the entire record of ‘*P.’ nestii* reveals that this species displays a mosaic of characters between *Dama* and *Cervus*, but also that the shared characters with *Dama* are prevalent (as already pointed out by Azzaroli^8^). The Pantalla sample allows to substantiate these conclusions very well. Our CT-based comparisons between the crania from Pantalla and those of extant red deer and fallow deer (Fig. 3) highlight some morphological similarities with the former, including a relatively longish neurocranium with steep forehead and deep preorbital fossa. On the other hand, *‘P.’ nestii* from Pantalla clearly shows *Dama*-like cranial characters, such as a marked interfrontal crest, horizontal zygomatic arch, high maxilla below the orbit, muzzle more inclined ventrally and less cylindrical in overall shape, sub-horizontal upper cheek tooth row (i.e., the occlusal margin of the row is approximately straight in buccal view), apical surface of the pedicle more inclined dorsocaudally, and overall morphology of the antlers, which in rostral view diverge, rather than converge as in the red deer (Fig. 2).

Likewise, the teeth from Pantalla, have a mixture of *Dama* and *Cervus* characters although the former are prevalent. All the premolar characters (the complete absence of a lingual grove on P4, the presence of a cingulum on the distolingual wall of P4, the presence of a small paraconid in p2, the entoconid more aligned with the mesiodistal axis in p3-p4, and a weak mesial cingulum on p4) and most of the lower molar characters are *Dama*-like. The upper molar features are instead more reminiscent of *Cervus* being either intermediate between the morphology of the latter and that of extant *Dama* or even matching *Cervus* (see Supplementary Table S6 and below).

The postcranial remains from Pantalla appear more similar to *Dama* than to *Cervus*. Of the 23 morphological characters by Lister^39^ which are present in the preserved bones (axis, metacarpal, tibia, astragalus, calcaneum, cubo-navicular, metatarsal, phalanx I, and phalanx II), 21 scores as fallow deer and only two as red deer (details in Supplementary Table S7).

A mixed character suite between *Dama* and *Cervus* are revealed also by our palaeoneurological analysis. The brain of *‘P.’ nestii* shows *Dama*-like size and *Cervus*-like morphology with a prominent cerebellum and a dorsoventrally flattened cerebrum. The latter character is clearly noticeable in *‘Pseudodama’* and *Eucladoceros*, is less evident in extant *Cervus*, and is missing in *Dama*. The hypothesis that depressed and longish cerebra represent a primitive character in Cervini (at least in Pleistocene European forms) is supported by our preliminary data and agree with Azzaroli^8^.

Most interestingly, the two crania from Pantalla actually show some remarkable morphological differences. The neurocranium of 337643 is more lengthened (i.e., more *Cervus*-like), albeit this shape might be taphonomically modified by the lateral compression of the specimen. This morphology fits that observed in some other *‘P.’ nestii* specimens such as IGF 1403 from Olivola (Italy), while the relatively shorter and more rounded neurocranium of 337655 resembles that of other specimens such as IGF 1404 also from Olivola. Moreover, 337643 shows a stronger nuchal crest than 337655. These differences may be related to ontogenesis (see the advanced age of 337643 based on tooth wear). In several cervid species including fallow deer, aging leads to morphological changes in the neurocranium, which tends to elongate and flatten and shows a more developed nuchal crest, probably as a response to the support of larger and heavier antlers^18,38^. Similarly, in 337643, the pedicles are apparently closer to one another due to their thickening—an expected condition for an old individual as the distance between the pedicles tends to decrease with age^8^—and markedly shorter than wide. Our comparative data on European *Dama*-like deer show that the pedicle section can be highly variable both within and between species, although a general trend of laterolateral flattening (i.e., oval shape with major axis oriented anteroposteriorly) can be traced through time (Supplementary Fig. S3), probably as a result of the development of wide, laterally-projecting palmated antlers (in extant deer, *D. dama* is among those with the heaviest antlers relative to body size^40,41^). Therefore, the Pantalla sample on the one hand confirms the variation in cranial morphology already observed for *‘P.’ nestii*^6,8^, on the other hand it supports the affinities between this species and the fallow deer. The presence of *Cervus*-like features especially in cranial morphology may be interpreted as plesiomorphic characters which, associated with some characters of the dentition and of the brain, suggest a basal position of *‘Pseudodama’* in the evolutionary history of the Cervini. This hypothesis may be tested in the future through phylogenetic analyses, currently made difficult by the lack of sufficiently well-preserved material of some species of *‘Pseudodama’* (e.g., *‘P.’ lyra*, *‘P.’ perolensis*).

Compared with other specimens of *‘P.’ nestii*^6,8^, the sample from Pantalla shows some plesiomorphic characters including a high ratio between the premolar and molar lengths, i.e., 0.77–0.82 (n = 2) for upper teeth (LP/LM) and 0.68–0.69 (n = 3) for lower teeth (Lp/Lm). These values are closer to the basal forms of *‘Pseudodama’*, such as *‘P.’ lyra* from Montopoli (LP/LM = 0.73, n = 1; Lp/Lm = 0.64, n = 2) and *‘P.’ rhenana* from Saint Vallier (LP/LM = 0.75, n = 9; Lp/Lm = 0.68, n = 18; data from Valli^42^), than to *‘P.’ nestii* from Olivola and Upper Valdarno (LP/LM = 0.72, n = 10; Lp/Lm = 0.63, n = 17). Other putatively plesiomorphic features of the sample from Pantalla are all those that approach it morphologically to *Cervus* (see Supplementary Table S6), i.e., the strong development of lingual conids and stylids in lower molars (Char. 4^39^) and of buccal cones and styles in upper molars (Char. 1^39^), the lack of a clear step between 2nd and 3rd lobe of m3 (Char. 11^39^), the strong lingual cingulum on upper molars (Char. 3^39^), and the lack of the horizontal turning of the buccal columns of upper molars (Char. 4^39^—the so-called buccal “cingulum”^43^). The strong lingual cingulum on upper molars is constantly present in the earliest species of the *‘Pseudodama’* group, *‘P.’ pardinensis*^9^, and still present, although extremely rare, in *‘P.’ lyra* from Montopoli, *‘P.’ rhenana* from Saint Vallier and Senèze, *‘P.’ perolensis* from Peyrolles, and *‘P.’ nestii* from Olivola. However, this feature is back less rare in *‘P.’ nestii* from Upper Valdarno and *‘P.’ farnetensis* from Selvella, suggesting a certain polymorphism at this stage. The lack of buccal “cingulum” is a constant in the earliest *‘Pseudodama’* populations (Lower Valdarno, Saint Vallier, Senèze), the buccal “cingulum” appearing, although rare, in *‘P.’ perolensis* from Peyrolles and *‘P.’ nestii* from Olivola and Upper Valdarno but becoming more common only in later *‘P.’ farnetensis*, *‘P.’ vallonnetensis*, and constant in *Dama*.

The above affinities between the Pantalla deer and the early representatives of *‘Pseudodama’* support the idea that the age of the assemblage may be close to the beginning of the Late Villafranchian (ca. 2.1–2.0 Ma), as already suggested based on the occurrence of *Leptobos merlai*^44^ and a primitive form of *Equus stenonis*^35^. Thus, the *‘P.’ nestii* sample described herein may represent one of the earliest occurrences of the species in Europe.

### Palaeoecological and palaeoethological inferences

The Pantalla sample is also noteworthy as it allows opening a window into the behaviour of these extinct deer. The anomalies found on the two male crania are probably the result of different traumas during their life.

Deer are well known for the intense fights they engage in during the rutting season using their antlers, as a result of an escalation of a broad repertoire of threats and displays^45^. Mineralized antlers are solid structures able to withstand the vehemence of the fight^46^, whereas growing antlers are extremely fragile and any contact with a solid object may result in a serious injury^47,48^ that may jeopardize the bearer’s ability to compete with conspecifics and, consequently, its dominance status^49^. Accidents are inevitable in the life of a deer and, in case of the suffered damage not leading to the breakage of the growing beam and consequent loss of its distal part, the antler may continue its growth although, in case of a severe lesion, at a crooked angle^45^. Thus, if the antler was just cracked and the broken part was held together by the velvet and periosteum, with the blood supply still being guaranteed, the damaged beam would just present a conspicuous swelling around the area of fracture (i.e., a fracture callus)^45,50^ and a change in the axis of orientation. These features match those seen in the left beam of 337655, which shows a fracture callus between the basal and middle tines corresponding to a change in the orientation of the beam.

The supernumerary tine of the right antler of the same individual can be interpreted as the result of a trauma, too. Considering the delicate nature of the growing antlers and the non-negligible risks of occurrence of an injury, it is safe to believe that the right antler has undergone a light traumatic event (most likely concerning the pedicle) at some early stage of its growth. In fact, it is known that limited injuries could result in the growth of supernumerary tines, even in atypical positions^51^, as it has been documented in other deer species (e.g., reindeer^52^, sambar^53^). It is therefore reasonable to hypothesize that both antler anomalies of 337655 derive from traumas suffered by the deer during the antler growth, when the velvet was still present. It is not known whether the two injuries happened at the same time or in two different events. In fact, it cannot even be said that the two events took place during the same season. While the breakage of the left beam must have occurred in the year of the animal’s death (i.e., during the velvet period preceding the period of hard antler in which the individual died), the development of the supernumerary tine on the right may be the result of a trauma suffered in a previous year. This is due to the fact that when unilateral trauma affects the generative region of the antler (i.e., the pedicle area), abnormalities such as supernumerary tines can reappear in next antler cycles even in more intensified forms^54^, as in the case of 337655 in which the extra-tine is extremely long.

The bone anomaly on the right squamosal of 337643 is also likely the outcome of an injury. Although the external portion was artificially smoothed during the preparation of the specimen, the outer and inner morphology matches that of a callus related to the healing of a major lesion and probable intracranial abscessation. Post-traumatic inflammatory processes are known to cause erosion or pitting of cranial bones in deer^55^ and can be triggered by many factors (e.g., wounds and abrasions of the pedicle^56^), among which violent sexual competition among males with hard antlers is considered one of the most common^55,57^. The advanced healing of the injury shown by 337643 suggests that it was not the cause of death, but rather that the individual survived a long time after the trauma albeit with the brain partially compressed by the callus.

**Figure 5:**
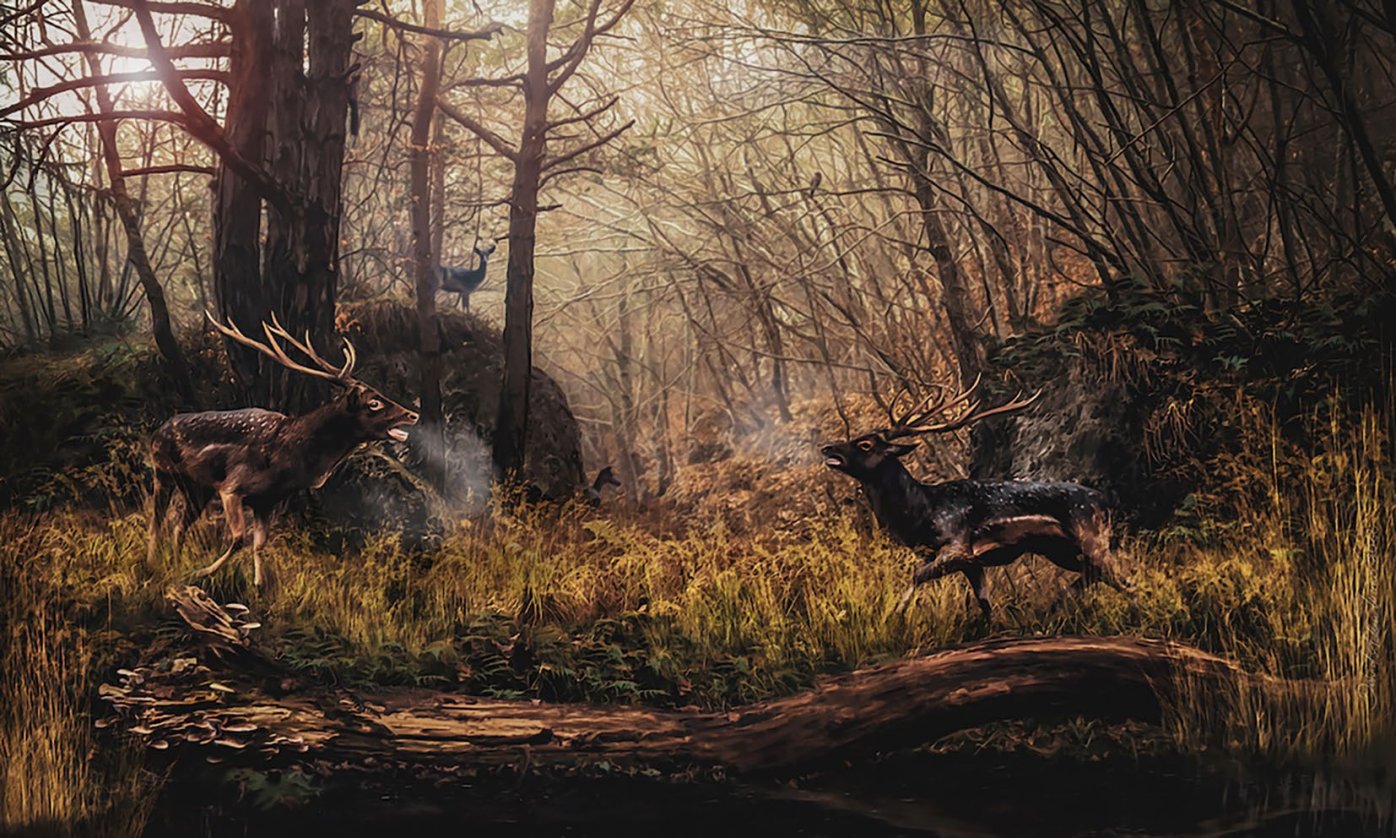
Life appearance of *‘Pseudodama’ nestii* represented during the rutting season. The reconstruction is based on the cranial and postcranial material from the Early Pleistocene of Pantalla (Italy) and on literature data. Artwork by D.A. Iurino.

The six mandibles recovered at Pantalla, all coming from the same bone accumulation hence reasonably referable to a single deer population, represent several age classes, from calves as young as a few months up to very old individuals (i.e., over 15 years; Supplementary Table S4). Unfortunately, no mandible can be safely associated with the two male crania, although 337631 may belong to the same individual as 337643 based on advanced wear and size. Interestingly, the three most significant cranial remains (crania 337655 and 337643 and frontal bone fragment with basal antler base 337625) belong to adult males, which probably died during the hard antler period (i.e., rutting season: 337655 and 337625) or shortly after (i.e., 337643). The absence of females (at least among the remains with certain sex attribution) contrasts with the population structure in the extant fallow deer, in which females represent on average 75% of the herd^58^. However, the relative abundance of males may increase up to 50% in the rutting season^59,60^. Therefore, in spite of the relatively low number of fossils available, based on the age and sex structure of the palaeopopulation and by analogy with the extant fallow deer, the most plausible hypothesis is that the Pantalla deer died during or immediately after the rutting season (Fig. 5).

## Methods

### Material and methods

The sample of *‘Pseudodama’ nestii* from Pantalla is kept in the Soprintendenza Archeologia, Belle Arti e Paesaggio dell’Umbria (Perugia; SABAP_UMB). The list of specimens is reported in Supplementary Table S2. Standard measurements (Supplementary Table S3) were recorded to the nearest 0.1 mm with a digital calliper. Comparative morphological (e.g., antlers: number of tines, angle of the basal tine, pedicle length and angle with respect to the neurocranium, palmation or lack of; teeth: cingulum on upper molars, molarization of P4) and morphometric (e.g., tooth measurements, ratio between the premolar and molar series and the total length of the cheek tooth row, measurements of the long bones and their articular facets, the ratio between the length of the metapodials and the size of their epiphysis) information were taken from the cited literature and from raw original data on several *Dama*-like deer from different European sites. Anatomical terminology mainly follows Heintz^9^ and Lister^39^.

### Locality

The palaeontological site of Pantalla is located about 30 km South of Perugia (central Italy; 42°52′46.79′′N, 12°24′23.26′′E). It represents one of the most interesting Late Villafranchian sites in Europe especially for the outstanding state of preservation of the remains, including some complete skulls of large mammals. Vertebrate fossils were unearthed from the uppermost part of the 15 m-thick section^34^, formed by an alternation of silty sand and clay layers with a maximum thickness of one meter each^34^. The succession is generally referred to the Early Pleistocene Santa Maria di Ciciliano Unit^34^, but a multidisciplinary project aimed at the stratigraphic and chronological characterization of Pantalla is still in progress. The majority of Pantalla vertebrate fossils come from a relatively small (ca. 2 m^2^) bone accumulation within a compact yellowish silty sand layer. Very few remains in this layer were found in anatomical connection. A smaller number of badly preserved remains come from multiple greyish clay layers stratigraphically above the main accumulation. Details on the stratigraphic provenance of the ‘*Pseudodama*’ remains described in this paper are reported in Supplementary Table S2. The Pantalla vertebrate assemblage is formed by an indeterminate bird (a single thoracic vertebra); Rodentia: *Apodemus dominans*; Carnivora: *Canis etruscus*, *Vulpes* sp., *Lynx issiodorensis valdarnensis*, *Acinonyx pardinensis*, *Lutraeximia umbra*; Artiodactyla: *Leptobos merlai*, ‘*Pseudodama*’ *nestii*, *Sus strozzii*; Perissodactyla: *Equus stenonis*; Proboscidea: *Mammuthus* cf. *meridionalis*^34,35,44,51–67^. This local faunal assemblage can be referred to the early Late Villafranchian (ca. 2.1–1.7 Ma) correlative with the Olivola-Tasso Faunal Units^68^ and MNQ 18 in the ‘Mammal Neogene/Quaternary Zones’ system^69^.

### Imaging

CT images of 337643 (slice number: 809) and 337655 (slice number: 647) were taken using a Fujfilm FTC Speedia 16-slice scanner at 220 mA and 120 kV, with slice thickness of 0.8 mm, interslice thickness of 0.4 mm, and voxel size equal to 0.7 mm. Those of the extant *D. dama* (CZUFLD FaD001, CZUFLD FaD002, CZUFLD FaD003; slice numbers: 913, 845, 566, respectively) and *C. elaphus* (CZUFLD ReD001, CZUFLD ReD002, CZUFLD ReD003; slice numbers: 1271, 1513, 719, respectively) were taken using a Siemens Somatom Scope Powerscanner, a multi-detector CT scanner with 16 slices at 240 mA and 130 kV, with both the minimal slice thickness and voxel size equal to 0.6 mm. For this study, the slice thickness was set to 1.0 mm and the recon increment to 0.5 mm. Moreover, we did a 3D multiplanar reconstruction to be able to see the X, Y, Z planes of the slices and we chose a hard algorithm (U 90 s) to highlight the bone density interfaces and achieve a higher resolution.

The segmentation process of the CT images was carried out with Mimics Innovation Suite 21.0, allowing the virtual extraction of the brain endocasts from crania and the acquisition of their biometric and morphological data. False colour filter was applied on the CT-images using the density filter tool available on Materialise 3-matic 13.0 part of the Mimics Innovation Suite 21.0. The final editing of the 3D images (e.g., colouring, transparency effect, and rendering process) was performed in ZBrush 4R6.

For some specimens, 3D models were built by *Structure-from-Motion* photogrammetry method and are available on https://www.morphosource.org (cranium 337643: media ID 000430779; cranium fragment 337625: media ID 000430794; mandible 337631: media ID 000430784). Pictures were processed under Agisoft PhotoScan Professional version 1.3 retrieved from agisoft.com/downloads/installer/. Technical specifications of the photogrammetric process are reported in MorphoSource.

## Supplementary Information

Supplementary Information.

## Data Availability

All data generated or analysed during this study are included in this published article and its supplementary information files, with the exception of raw data used to build Supplementary Fig. S3, which are available from the corresponding author on reasonable request. 3D models of three specimens are available on the MorphoSource on-line repository (https://www.morphosource.org; media IDs: 000430779, 000430794, and 000430784).

## Abbreviations

CZUFLD: Czech University of Life Sciences Prague, Faculty of Forestry and Wood Sciences (Czech Republic)
IGF: Museo di Storia Naturale, Sezione di Geologia e Paleontologia, Università di Firenze (Italy)
SABAP_UMB: Soprintendenza Archeologia, Belle Arti e Paesaggio dell’Umbria (Italy)
