# Supplementary material for: A Pleistocene Fight Club revealed by the palaeobiological study of the Dama-like deer record from Pantalla (Italy)
Source: Sci Rep. 2022 Aug 16;12:13898. doi: 10.1038/s41598-022-18091-1 (PMC9381596; doi:10.1038/s41598-022-18091-1)
Supplement: Supplementary file 1 — Supplementary Information. [file 41598_2022_18091_MOESM1_ESM.pdf]

Supplementary Information

A Pleistocene Fight Club revealed by the palaeobiological study of the *Dama*-like deer record from Pantalla (Italy)

Marco Cherin, Marzia Breda, Bruno Esattore, Vlastimil Hart, Jiří Turek, Francesco Porciello, Giovanni Angeli, Sofia Holpin & Dawid A. Iurino

Summary

Supplementary Note 1	Pag. 2
Supplementary Figures	Pag. 9
Supplementary Tables	Pag. 12
Supplementary References	Pag. 30

Supplementary Note 1
Description of the material of *'Pseudodama' nestii* from Pantalla (Italy)

Cranium

SABAP_UMB 337643 (the abbreviation SABAP_UMB is omitted hereinafter) is a well-preserved un-antlered male cranium of an adult individual, with complete right cheek tooth row (Fig. 1c). The isolated P2 167344 has been associated to the cranium. All teeth (especially M1) show a very advanced state of wear. The left maxilla, lower orbit, and zygomatic arch are missing and the muzzle is broken off just before the level of P2. The temporal fossa is longitudinally elongated and rhomboidal in shape. The parietal and frontal dorsal outlines form an obtuse angle at the fronto-parietal suture. Rostrally to the pedicles, the frontals slope steeply forming a sharp forehead, which rises obliquely above the orbit. Caudally, the parietal bones slope back gently. The right zygomatic arch appears very thin and sub-horizontal. The postorbital rim constitutes a strong posterior orbital margin. The right orbit is rounded and two lacrimal foramina are visible on its rostral margin. Rostrally to the orbit, a deep and triangular preorbital fossa is present. The ethmoidal vacuity is separated from the preorbital fossa by a sharp crest, but is filled with sediment. It is slightly larger than the preorbital fossa. In dorsal view, the supraorbital foramina are located at about mid-length of the orbit. The frontals are slightly concave in the area between the supraorbital foramina and the base of the pedicles. Conversely, they appear convex between the orbits and especially between the pedicles, where –in correspondence with the sagittal suture– they form an elevated and massive crest. The pedicle transverse section is oval, with the longer axis perpendicular to the sagittal plane. The pedicles diverge from each other. The parietal roof is almost flat, but a squared prominent area exists in the nuchal interparietal area, connected to the nuchal crests. In caudal view, the foramen magnum is almost circular. The occipital squama is high and sub-trapezoidal in shape. It is divided by a strong vertical median bulge running ventrally from the prominent external occipital protuberance. Laterally to this median bulge, the occipital squama is markedly concave and delimited dorsolaterally by prominent nuchal crests. In ventral view, the palate ends at the level of M3's distal edge. The palatine laminae are both preserved and delimit narrow choanae laterally. The palatine foramina are obliterated by sediment. The glenoid cavities are wide and convex. The basisphenoid is rostrally pointed and delimited laterally by high and sharp pterygopalatine crests. Laterally to the occipital tubercles, the tympanic bullae are relatively small and nasally pointed. The basioccipital is wide and almost flat. Two pointed basilar processes are visible on the two caudolateral corners of the basioccipital. The jugular processes are broken. A small and laterally pointed mastoid process is preserved on the right side.

Specimen 337655 (Fig. 1a) consists of a nearly complete male neurocranium with exceptionally completely preserved antlers. The right maxillary fragment with P3–M3 337633 (Fig. 1d) and the left maxillary fragment with P2–P4 337637 (Supplementary Fig. S1e) might be associated to the same individual based on similar fossilization features. Similarly, due to

perfect occlusion with 337633 and same taphonomic appearance, also the right hemimandible with p2–m3 337630 (Supplementary Fig. S1) might belong to the same skull. The morphology of the neurocranium and antlers of 337655 are described in the main text.

Upper teeth

The upper cheek teeth can be fully observed and described in the maxillae 337633 (right fragment with P2–M3; Fig. 1d) and 337637 (left fragment with P2–P4; Supplementary Fig. S1b) thanks to the excellent preservation. In occlusal view, P2 and P3 are similar in size and general morphology. Both exhibit an incipient molarization, that is, a lingual notch (deeper in P2) separating the tooth into a mesial and a distal lobe (protocone and hypocone, respectively). The parastyle, mesostyle, and especially metastyle have pointed margins. On the contrary, P4 is shorter, mesiodistally compressed, and with less pointed labial styles. Distolingual cingula are visible on P3 and P4. In all premolars, the central cavities are well developed and show enamel folds projecting especially from the lingual side (e.g., see the three-chambered appearance of the central cavity of P2 in 337633; Fig. 1d). The upper molars are also mesiodistally compressed, thus showing an overall squared occlusal outline. The mesiolingual, distolingual, and especially lingual cingula are strong in all molars, particularly in M3. The lingual cingulum culminates in the middle with a well-developed entostyle. Buccally, the cusps and styles are very projecting. The same overall morphology is observable in all other preserved upper teeth from Pantalla.

Mandible

The most complete mandibular remain is 337631 (Supplementary Fig. S1g), a left hemimandible still fused to the symphyseal portion of the right corpus. The specimen only misses the tip of the coronoid process and the ventral margin of the angular process. In labial view, the corpus is very elongated and slender, with a ventral margin only slightly undulated. The diastema is particularly long. The angular process does not protrude much caudally and is quite squared, although this shape may be exaggerated by erosion of the ventral margin (in fact, a slightly more rounded process is visible in 167339). The ramus rises nearly vertical and does not seem to bend posteriorly caudally even above the condyle. On the lingual side, a circular mandibular foramen opens at about the occlusal level of the lower teeth.

Lower teeth

All lower cheek teeth, especially the molars, exhibit a rough lingual surface. In the right hemimandible 337630 (Supplementary Fig. S1e), the mesial crest of p2 is mostly formed by a strong parastylid but shows also an incipient paraconid, not visible in the other specimens. The mesial crest is separated from the metaconid by a deep and narrow second valley (*sensu* Heintz[9]). In p3, the second valley is wider than the third due to the backward orientation of the metaconid. In p4, the second valley is closed by the connection between paraconid and metaconid and is replaced by an enamel islet in the middle of the mesial lobe of the tooth; the

third valley is shaped as a very shallow incision not visible along the lingual wall. The distal part of p4 is wide mainly due to the development of a massive hypoconid. Lower premolars do not show cingula in any specimen. Conversely, a strong mesiobuccal cingulum is present in all lower molars. These latter also show well-developed but low ectostylids between mesial and distal lobes and, in m3, also between distal lobe and hypoconulid. In little worn molars, a V-shaped opening on the lingual side between entostylid and posterior hypoconid wing is present. In 337631 (Supplementary Fig. S1g), although left and right i1 are displaced by diagenesis, the teeth forming the incisor arcade are arranged along a wide arch. The labial wall of i1 is markedly bent outwards.

As for deciduous teeth (Supplementary Figs S1c and S1f), dp2 is a short and narrow tooth, with sub-triangular occlusal outline; it mimics the general morphology of p2 but has a shallower second valley. On the contrary, dp3 is relatively long and narrow, with poorly developed first valley and deep second valley. The dp4 is formed by three similar-sized lobes, between which low and pointed ectostylids rise up, similar to those observed in permanent molars.

Vertebrae

Specimen 337649 comprises a well-preserved axis (Supplementary Fig. S2a), cervical vertebrae III–IV (connected by sediment), and three articulated caudal vertebrae.

In the axis, only the anterior portion of the spinous process is preserved and it projects cranially (as in *D. dama*, Char. 4[39]), but not further than the dens. Both transverse processes are destroyed along with the left side of the anterior articular facet and both postzygapophyses. The left portion of the body is also damaged. Of the cranial transverse foramen, only the dorsal opening is visible as it is fused with the intervertebral foramen. The dens protrudes more than the articular facet with whom it produces a right angle. On the right side, the vertebral body is mostly intact along with the rim of the cranial articular facet and the ventral crest. In anterior view, the dens is damaged on the right margin, but otherwise once formed a single, crescent-shaped articular facet. The neural arch is entirely filled with sediment.

The cervical vertebra III (better preserved than the IV) presents a tear-drop-shaped cranial articular surface that is convex and widest at its dorsal margin. The spinous process is missing, along with most of the transverse processes and tubercles. In dorsal view, the left prezygapophysis is intact. The ventral tubercle of the left transverse process is also partially present. The two postzygapophyses are damaged, but their general outline is still recognizable. In caudal view, the caudal articular surface is concave and mostly broken. Dorsally, the neural arch delimits a large canal, which is narrower at its ventral margin. On either side, the transverse foramina are less noticeable than on the cranial margin.

Metacarpal

Specimen 337652 (Supplementary Fig. S2b) is a right metacarpal missing the distal portion.

The section of the diaphysis is D-shaped. The dorsal surface is strongly convex whereas the lateral and medial surfaces are flatter. The tuberosity at the proximomedial corner of the anterior surface is not rough (as in *D. dama*, Char. 5[39]). The palmar surface is characterized by a central groove – mostly destroyed. A clearly-defined mild pit is visible at the proximomedial corner of the palmar surface (as in *D. dama*, Char. 4[39]). The proximal epiphysis is intact. The articular surface is trapezoidal in outline – the dorsal margin is rounded whereas the medial and palmar margins are straighter. The articular surface is smooth. The medial articular facet is larger than the lateral one, and the two are joined palmarly (as in *D. dama*, Char. 1[39]). They surround a large nutrient foramen, which is oval in shape and located just before the palmar margin. The medial articular facet is subrectangular, with rounded edges. Contrarily, the lateral articular facet is subtriangular.

Hindlimb

Specimen 167353 is a right partial articulated hindlimb composed of a distal tibia, astragalus, incomplete calcaneum, lateral malleolus, cubo-navicular, cuneiforms, metatarsal with medial phalanx I and sesamoids attached by sediment, lateral phalanges I and II, and lateral sesamoids (Supplementary Fig. S2d-m). The medial phalanx II 167357 is almost certainly part of the same limb.

The tibia (Supplementary Fig. S2d) is broken approximately half way up the shaft, thus missing the proximal portion whereas the distal epiphysis is intact. The diaphysis, where preserved, presents an oval section – dorsoplantarly compressed. In dorsal view, the shaft is slightly concave and flat, just above the epiphysis. Plantarly it is convex. The medial malleolus is slender and extends below the central spine – it also presents a small articular facet on its lateral surface. In distal view, the articular surface is intact and well preserved – having been fossilized articulated with the other elements of the ankle. Both the articular grooves are visible; the medial one is more mediolaterally compressed and presents narrow dorsal and plantar margins. The lateral articular groove is more rounded in outline and is separated from the medial groove by a prominent ridge. They are both parallel to the longitudinal axis of the shaft. On the lateral margin of the articulation, there are two small articular surfaces – slightly concave and rounded in shape. The dorsal articular facet is smaller, downturned plantarly (as in *D. dama*, Char. 4[39]), and separated from the plantar facet by a deep V-shaped groove. Dorsally to the dorsal articular facet a small flat shelf develops (as in *D. dama*, Char. 3[39]). In medial view, the medial malleolus appears oriented dorsally, describing a wide U-shaped dorsal margin (as in *D. dama*, Char. 5[39]). Plantarly to the medial malleolus there is a groove that extends up the shaft, parallel to it. This groove is also visible in plantar view.

The astragalus (Supplementary Fig. S2i) is well preserved. In dorsal view, the proximo-lateral condyle protrudes more than the medial one. The central portion forms a flat-bottomed U-shape (as in *D. dama*, Chars. 1-2[39]). The lateral projection of the lateral condyle has a vertical margin (as in *D. dama*, Char. 4[39]). The lateral bulge between the proximal and distal half of the astragalus is pointed and projects laterally in dorsal view (as in *C. elaphus*, Char.

5[39]). Both trochlear condyles are parallel to the sagittal plane and the surface in between is strongly concave. Plantarly, the articular facet for the calcaneum is proximo-distally convex and occupies most of the plantar surface. Proximo-laterally to the facet, the ridge of the lateral condyle is triangular in shape and pointed, with a rounded furrow on its lateral surface. On the distal portion, there are two small oval depressions, which respectively articulate with the medial and central proximal projections of the cubo-navicular. In medial view, the distal condyle is rounded. The dorsomedial corner of the medial surface strongly projects dorsally (as in *D. dama*, Char. 3[39]). The proximal portion presents a well-defined furrow along its dorsal margin – where the medial malleolus articulated – on its plantar margin it ends in a pointed protuberance. The remaining medial surface appears rough. In lateral view, the distal border of the lateral condyle is slightly convex and corresponds to the calcaneum articular facet. The space in between the two is irregular.

The calcaneum (Supplementary Fig. S2j) misses almost all the dorsal process, as only the articular facet to the lateral malleolus and part of the plantar articular facet for the cubo-navicular are preserved. In distal view, the main articular surface is squared in outline, with rounded dorsal and medial margins. The body of the calcaneum is intact. The proximal portion of the body is elongated, medio-laterally compressed, and presents an irregular, enlarged *tuber calcanei.* The trochlear facet – a rounded projection that articulates to the lateroplantar margin of the astragalus – and the plantar margin of the main articular surface are visible. Medially, the trochlear facet appears semi-circular in outline. It is connected to the main articular facet by a small, concave articular surface. The *tuber calcanei* in medial view presents a furrow, that separates the plantar portion and the dorsally pointed portion. The dorsal and plantar borders are straight and parallel to the proximodistal axis. The *sustentaculum tali* protrudes medially and the plantar end is inclined distally. It has an oval outline and does not protrude beyond the plantar margin of the main body – between the two there is a deep groove. The scar on the plantar surface extends further than the dorsal edge of the *sustentaculum tali* (as in *D. dama*, Char. 1[39]).

The cubo-navicular (Supplementary Fig. S2k) is almost perfectly preserved, a portion of the dorsal medial margin is broken and there is sediment adhering to the articular surfaces and to the plantar margin. In proximal view, the articular surface has two grooves parallel to the sagittal plane. Plantarly to each groove, there is a pointed projection – the medial one larger than the lateral one. On the lateral margin, there is an elongated, convex articular surface that articulated with the dorsal process of the calcaneum. Distally, the four articular facets are intact. The general outline is that of a quadrilateral with rounded edges and the dorsal articular facets are larger than the plantar ones. The larger, dorsolateral facet for the metatarsal is separated from the others by an L-shaped non-articular surface. At the junction, there is a small pit. The small, plantar articular facet for the metatarsal is orientated mediolaterally and has an elongated shape. The medioplantar facet for the small cuneiform is bean shaped. In plantar view, the medial and lateral projections are most noticeable. The furrow separating them is also visible and is V-shaped with rounded margins. The lateral

projection of the calcaneum facet is lower than the medial projection. Laterally to the groove there is a large foramen almost directly in the centre of the plantar wall. The overall outline is rectangular.

The large cuneiform (Supplementary Fig. S2g) is proximo-distally compressed. The proximal surface is concave, while the distal one is convex. The plantodistal margin is narrow and pointed. The small cuneiform (Supplementary Fig. S2g) is rounded and drop shaped. It also is proximo-distally compressed.

The lateral malleolus (i.e., the vestigial distal portion of the fibula - Supplementary Fig. S2f) is irregular in shape. Proximally it articulates to the tibia, medially to the astragalus, and distally to the calcaneum.

The metatarsal (Supplementary Fig. S2e) is quite well preserved – two sesamoid bones and the medial phalanx I are still articulated, attached by sediment. The diaphysis is broken in numerous places, causing the distal epiphysis to bend medially. The diaphysis is damaged mainly in the central part, where the walls of the shaft are mediolaterally compressed along the mid-line groove and have crashed into the medullary cavity. The phalanx leans ventrally. The proximal epiphysis is rectangular in outline and the nutrient foramen is visible. The four articular facets are smooth; the dorsal two are concave whereas the plantar two are coated in sediment. The dorsomedial and dorsolateral facets join each other dorsally (as in *D. dama*, Char. 1[39]) and lie on the same level (i.e., there is not a step between the two (as in *D. dama*, Char. 2[39]). The distal epiphysis is coated in sediment and – in lateral view – leans slightly ventrally. In plantar view, the two sesamoid bones are still in anatomical position, on the plantar surface of the medial trochlear condyle. Looking at the proximal edge of the intertrochlear groove in plantar view, no split is visible (as in *D. dama*, Char. 5[39]). The overall morphology of the right metatarsal 337651 (Supplementary Fig. S2c) is very similar to that of 167353, although the state of preservation of the proximal articular surface is quite poor in the former.

The medial phalanx I of 167353 (Supplementary Fig. S2e) is still articulated to the medial condyle of the metatarsal, while the lateral phalanx I is disarticulated. These phalanges are almost complete, with the exception of the proximo-lateral edge of the medial one. In proximal view, there is not a step-like projection of the groove between the articular facets (as in *D. dama*, Char. 1[39]). In the lateral phalanx, the medial metapodial facet is somewhat more dorsoplantarly extended than the lateral metapodial facet. The latter also protrudes more with respect to the medial facet. On the plantar margin of the articular surfaces, there are two rounded sesamoid facets. The plantar end is rectangular, in outline, with rounded edges.

The medial phalanx II (Supplementary Fig. S2m) is slightly larger than the lateral one (167357), but overall, very similar in morphology. In lateral view, the proximal end is deeper than the distal one thus the outline of the bone is subtriangular. The dorsal and plantar walls of the diaphysis are straight. The distal end is rounded in outline. In plantar view, the distal and proximal ends are aligned and present a groove along the axial plane. On the proximal

end, the lateral tubercle is larger than the medial tubercle. The dorsomedial edge of the proximal surface shows a concavity (as in *D. dama*, Char. 1[39]). At the distal end, the medial and lateral articular surfaces are smooth and lean inwards towards the sagittal groove.

Supplementary Figures

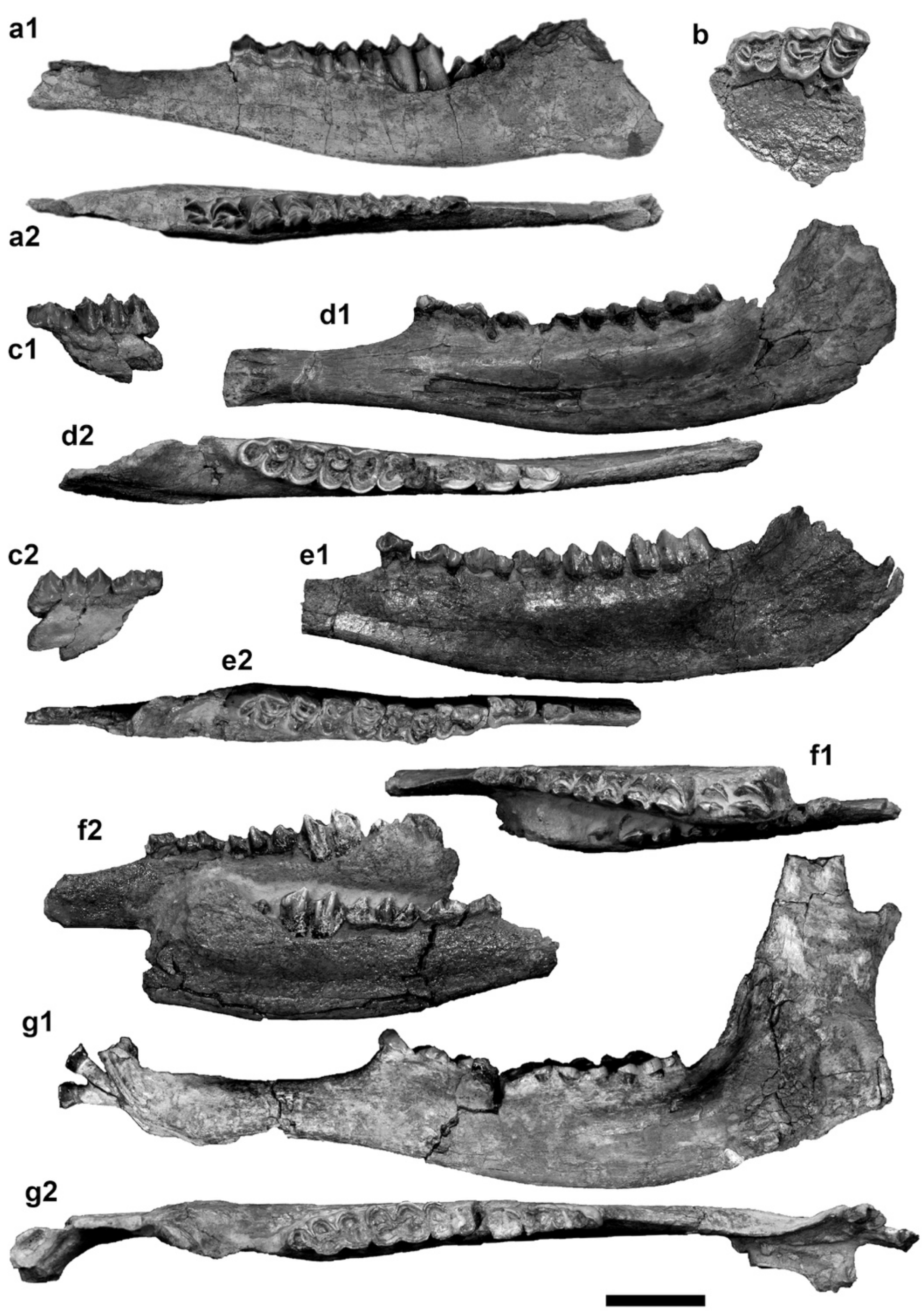

Supplementary Fig. S1. Selected mandibular and dental remains of *'Pseudodama' nestii* from Pantalla (Italy). (**a**) Left hemimandible with dp2-m1 and erupting m2 SABAP_UMB 167339 in labial (**a1**) and occlusal (**a2**) views. (**b**) Left maxillary fragment with P2–P4 SABAP_UMB 337637 in occlusal view. (**c**) Left hemimandible fragment with dp3-dp4 SABAP_UMB 167356 in labial (**c1**) and lingual (**c2**) views. (**d**) Right hemimandible with p2-m3 SABAP_UMB 337629 in lingual (**d1**) and occlusal (**d2**) views. (**e**) Right hemimandible with p2-m3 SABAP_UMB 337630 in lingual (**e1**) and occlusal (**e2**) views. (**f**) Left and right associated mandible fragments with dp2-m1 and erupting m2 SABAP_UMB 337632 in occlusal (**f1**) and left lingual (**f2**) views. (**g**) Left hemimandible with complete cheek tooth dentition, roots of i2-c1, and partial symphysis including a fragment of the right hemimandible. SABAP_UMB 337631 in labial (**g1**) and occlusal (**g2**) views. Scale bar: 3 cm.

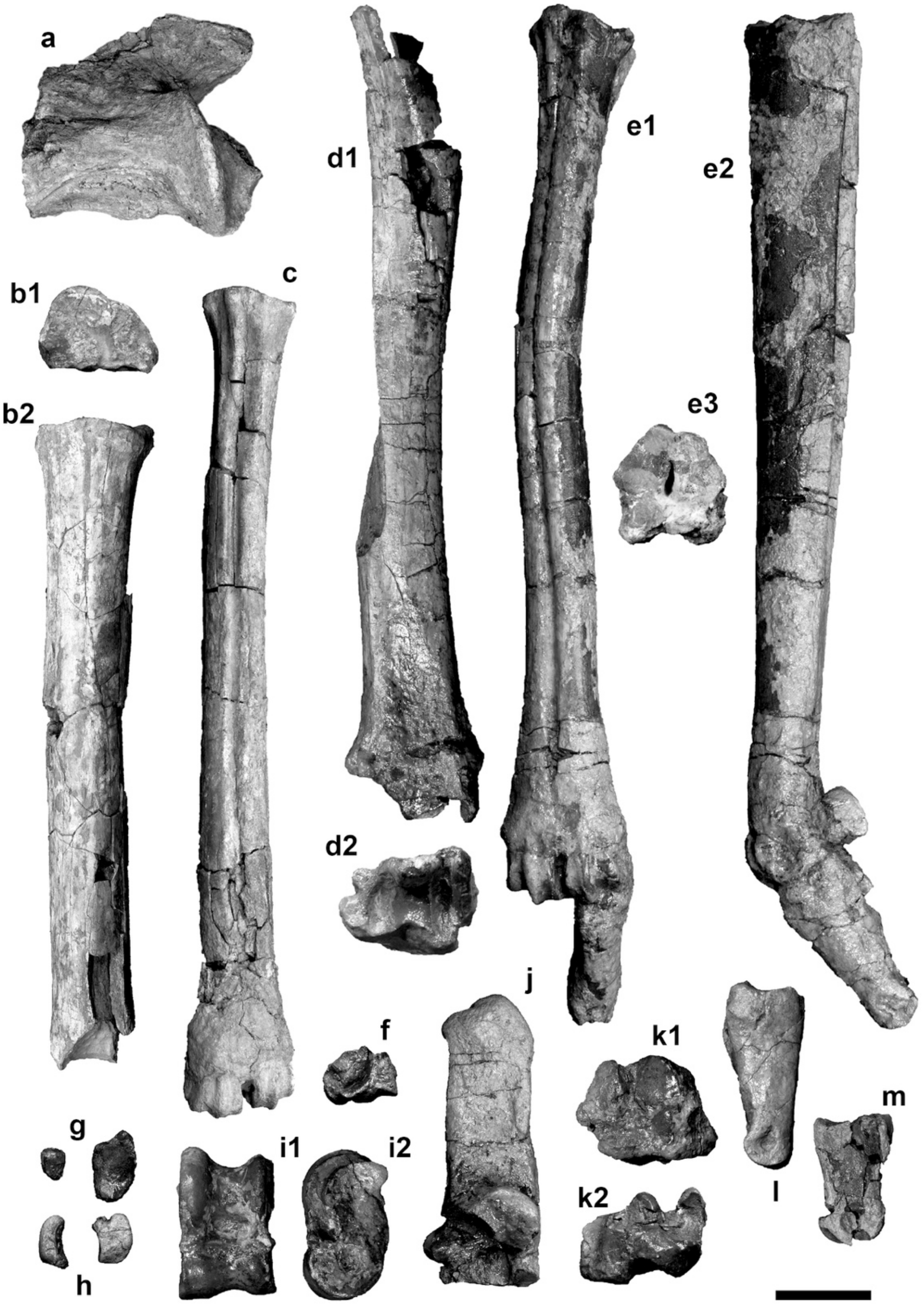

Supplementary Fig. S2. Selected postcranial remains of *'Pseudodama' nestii* from Pantalla (Italy). (**a**) Axis SABAP_UMB 337649 in right lateral view. (**b**) Fragmented right metacarpal. SABAP_UMB 337652 in proximal (**b1**) and anterior (**b2**) views. (**c**) Right metatarsal, SABAP_UMB 337651 in dorsal view. (**d-m**) Right articulated hindlimb SABAP_UMB 167353: (**d**) tibia in dorsal (**d1**) and distal (**d2**) views; (**e**) metatarsal with articulated medial phalanx I and sesamoids in dorsal (**e1**), medial (**e2**), and proximal (**e3**) views; (**f**) lateral malleolus in medial view; (**g**) small and large cuneiforms in proximal view; (**h**) two sesamoids in lateral view; (**i**) astragalus in dorsal (**i1**) and medial (**i2**) views; (**j**) calcaneum in medial view; (**k**) cubo-navicular in distal (**k1**) and dorsal (**k2**) views; (**l**) lateral phalanx I in abaxial view; (**m**) medial phalanx II in abaxial view. Scale bar: 3 cm.

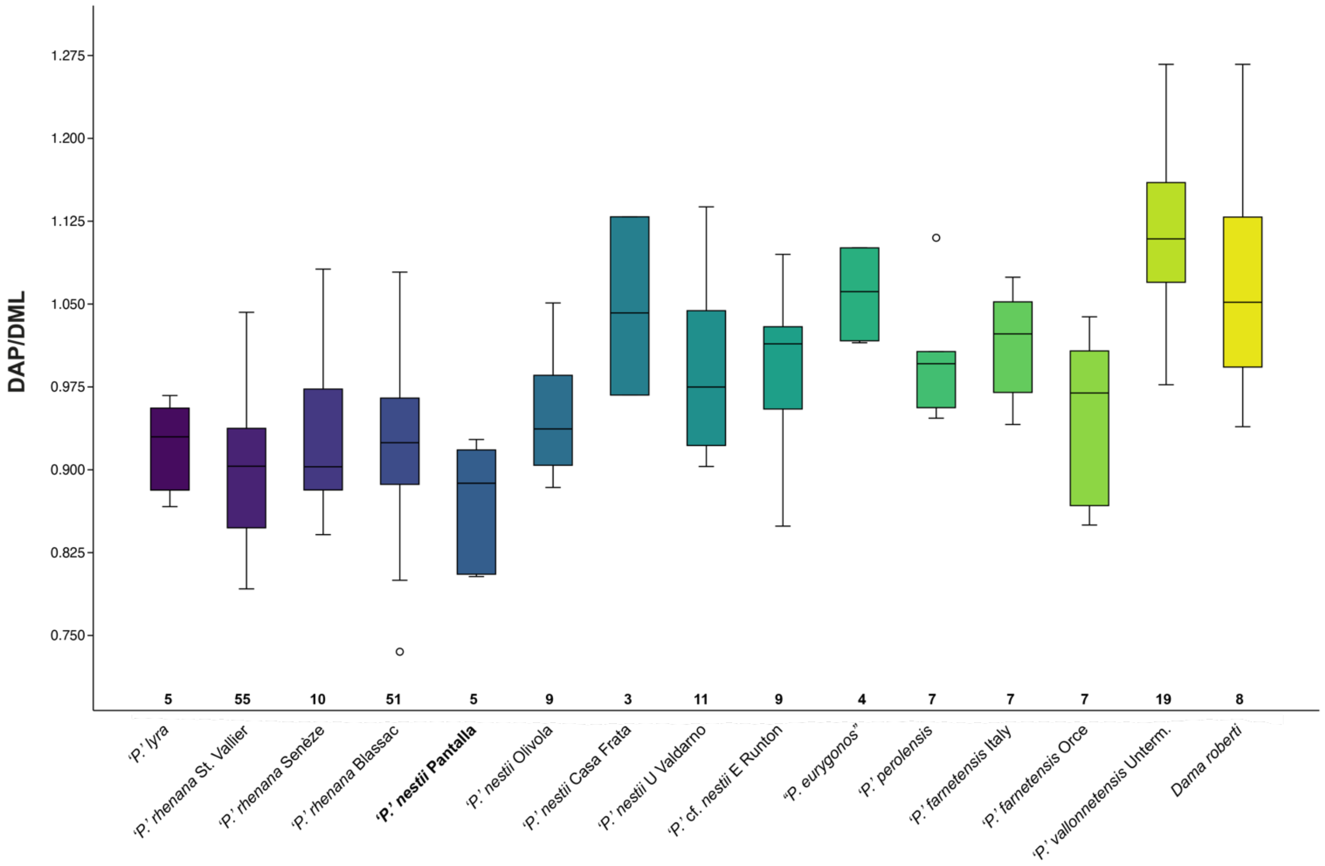

Supplementary Fig. S3. Boxplot of the ratio between the anteroposterior diameter (DAP) and mediolateral diameter (DML) of the pedicle in several *Dama*-like deer samples from the Plio-Pleistocene of Europe, shown in approximate chronological order (the sample size is shown above the labels). A general tendency towards mediolateral compression of the pedicle over time is observed.

Supplementary Tables

Supplementary Table S1. List of Plio-Pleistocene European sites bearing remains of *'Pseudodama'*. Dubitative records are indicated by ? in the first column. Unless otherwise specified, the taxon indicated in the Notes column is the one used in the primary reference. The list for *'Pseudodama' nestii* includes *"Dama nestii eurygonos"* (*sensu* Azzaroli[8]), considered as belonging to *P. nestii* by Azzaroli[6]. Specimens referred to *"eurygonos"* can be actually included into the variation of *'P.' nestii*. However, the type of *"Dama nestii eurygonos"* (IGF 245) from the Upper Valdarno (unknown locality) shows affinities with *'P.' farnetensis* (e.g., basal tine placed just over the burr and forming an open angle with the beam; middle tine relatively short; distal fork arranged in a parasagittal plane).

	Site	Country	Biozone	Faunal Unit	Age	References	Notes
'Pseudodama' pardinensis							
	Capeni	Romania	MNQ 15b			[1]	*Metacervoceros* cf. *pardinensis* (only faunal list)
	Varghis	Romania	MNQ 15b			[1]	*Metacervoceros* cf. *pardinensis* (only faunal list)
	Araci - Fântâna Fagului Quarry	Romania	MNQ 16a	Triversa		[1]	*Metacervoceros pardinensis* (only faunal list)
	Vialette	France	MNQ 16a	Triversa	ca. 3.14	9,[2]	*'Cervus' pardinensis*
	Hajnáčka	Slovakia	MNQ 16a	Triversa	ca. 3.0?	[3]	*Metacervoceros pardinensis* - No crania/antlers, but developed lingual cingulum on upper molars
	Covrigi	Romania	MNQ 16a	Triversa		[1]	*Metacervoceros pardinensis* (only faunal list)
	Grosera	Romania	MNQ 16a	Triversa		[1]	*Metacervoceros* cf. *pardinensis* (only faunal list)
	Cernatesti	Romania	MNQ 16a	Triversa		[1]	*Metacervoceros pardinensis* (only faunal list)
	Perrier-Les Etouaires	France	early MNQ 16b	Triversa	ca. 2.78	6,9	*"Cervus" pardinensis*
	Crouzas	France	MNQ 16	Triversa	ca. 3.1	[4]	*"Cervus" pardinensis*
	Farnier	France	MNQ 16	Triversa	ca. 3.0?	[4]	*"Cervus" pardinensis*
	Aulla Quarry	Italy	MNQ 16	Triversa		[5]	*Pseudodama* ex gr. *pardinensis-lyra* - Basal portion of shed antler with fragment of basal tine

'Pseudodama' lyra							
	Ponte a Elsa	Italy	MNQ 16a	Triversa		6	
	Collepardo	Italy	MNQ 16a	Triversa		[6]	
cf.	Spicchio	Italy	MNQ 16b	Montopoli	?	6	
cf.	Montopoli	Italy	late MNQ 16b	Montopoli	ca. 2.6	6	
	Valle Catenaccio	Italy	late MNQ 17b	Coste San Giacomo		[7]	*Axis lyra*
***'Pseudodama' rhenana* (= *'Pseudodama' philisi*)**							
	Pardines	France	early MNQ 17a	Saint Vallier	ca. 2.6	9	No cranium/antler remains
cf.	Varshets	Bulgaria	early MNQ 17a	Saint Vallier	ca. 2.6	[8]	
	Saint Vallier	France	early MNQ 17a	Saint Vallier	ca. 2.5	9,42,[9]	
	Zadar Archipelago (Iž, Rutnjak, Rava)	Croatia	MNQ 17a	Saint Vallier		[10]	*Cervus* cf. *philisi* and *Dama* sp.
	Volax	Greece	late MNQ 17a	Saint Vallier (?)	ca. 2.3	[11,12]	No cranium/antler remains
	Dafnero	Greece	late MNQ 17a	Saint Vallier (?)	ca. 2.3	[12]	No cranium/antler remains
cf.	Vatera	Greece	late MNQ 17a	Saint Vallier (?)	ca. 2.3	[13]	No cranium/antler remains - *Dama* cf. *rhenana*
aff.	Sesklo	Greece	late MNQ 17a	Saint Vallier (?)	ca. 2.3	[12]	No cranium/antler remains
	Chilhac II	France	early MNQ 17b	Saint Vallier	> 2.36	9,[14]	*Cervus philisi*
	Le Coupet	France	early MNQ 17b	Saint Vallier	2.36–2.21	9	No cranium/antler remains
	Šandalja 1 (near Pula, Istria)	Croatia	MNQ 17b	Saint Vallier		[10]	*Cervus philisi* and *Dama nestii*
	Cornillet (Moustiers-Sainte-Marie)	France	MNQ 17	Saint Vallier		[15,16]	No cranium/antler remains - One m3 referred to *Cervus* cf. *philisi*; two m3, upper teeth and one phalanx referred to *Dama nestii*
	Valea Graunceanului	Romania	MNQ 17			[1]	
cf.	Coste San Giacomo	Italy	late MNQ 17b	Coste San Giacomo	ca. 2.1	[17]	No cranium/antler remains - *Axis lyra* (= *C. philisi*)[18]

cf.	Almenara-Casablanca 1	Spain	late MNQ 17b (?)	Coste San Giacomo (?)	ca. 2.1 (?)	[19]	No cranium/antler remains - *Cervus philisi*[20], *Pseudodama nestii*[21,22]
cf.	Cava Toppetti	Italy	late MNQ 17b	Coste San Giacomo	ca. 2.1	[23]	
	Senèze	France	early MNQ 18		2.21–2.09	9,[24]	
	La Puebla de Valverde	Spain	early MNQ 18	Coste San Giacomo (?)	2.14–1.95	9,[25]	
	Erpfingen Höhle (= Erpfingen 2)	Germany	MNQ 18b?	Coste San Giacomo	ca. 2.0-2.1	[26]	*Cervus rhenanus/philisi* group
	Montoussè 5	France	early MNQ 18	Coste San Giacomo (?)	ca. 2.2	13,[27]	*"Cervus" philisi*
aff.	Gerakarou	Greece	early MNQ 18	Olivola	ca. 2.0-1.8	[12]	No cranium/antler remains
	Fonelas P-1	Spain	early MNQ 18	Olivola (?)	ca. 2.0 (?)	[28,29]	*Metacervoceros rhenanus philisi*[28], *Metacervoceros rhenanus perolensis*[29]. The fauna is probably older (or mixed).
	Blassac-la-Girondie	France	mid MNQ 18 (?)	Olivola (?)	ca. 2.0–1.8 (?)	[22]	*Cervus ischnoceros*[30], *Cervus perolensis*[31], *"Pseudodama" rhenanus perolensis*[13]
?	Slivnitsa	Bulgaria	mid MNQ 18	Olivola	ca. 1.9	[8]	*Cervus rhenanus* - *Pseudodama nestii*
	Nogaret	France	mid MNQ 18	Olivola (?)	ca. 1.9-1.8	[32]	No cranium/antler remains
	Tegelen	The Netherlands	late MNQ 18	Tasso (?)	ca. 1.8	[33]	
	Oosterschelde	The Netherlands	late MNQ 18 (?)	Tasso (?)	ca. 1.8 (?)	[34]	No antler remains, only two upper tooth rows
	Strmica	Croatia	MNQ 18	Olivola-Tasso		[10]	*Cervus philisi*
?	East Runton	UK	MNQ 18	Olivola-Tasso	ca. 1.9–1.7	[35]	*Cervus rhenanus*[35], *Dama nestii nestii*[36]
	Triotra Vryssi	Greece	late MNQ 18	Tasso-Farneta	ca. 1.8-1.5	[37]	*"Cervus" philisi*
?	Denizli	Turkey	late MNQ 19	Pirro (?)	> 1.2	[38]	*Metacervoceros rhenanus* - Open angle between first tine and beam; quite high first tine. *Pseudodama* sp.?
	Ceyssaguet	France	late MNQ 19 (?)	Pirro (?)	ca. 1.2 (?)	21	*Metacervoceros rhenanus* + *Dama* cf. *vallonnetensis*
?	Saint-Privat d'Allier	France	MNQ 18-19		ca. 1.8-1.2	[39]	*"Cervus"* sp. (=*Pseudodama* p.p. sp.), similar to *"Cervus" rhenanus*
'Pseudodama' nestii							
	Olivola	Italy	mid MNQ 18	Olivola	ca. 2.0	6,8	

	Pantalla	Italy	mid MNQ 18	Olivola	ca. 2.0	This paper	
	Torre Picchio	Italy	mid MNQ 18	Olivola	ca. 1.9	[40]	
	Upper Valdarno (lower part: Faella, Matassino, Poggio Rosso)	Italy	mid MNQ 18		ca. 1.95-1.83	6,8,[41]	
	Podere San Lorenzo	Italy	mid-late MNQ 18	Olivola-Tasso	ca. 2.0-1.8	29	
	Casa Frata	Italy	late MNQ 18	Tasso	ca. 1.8	[42]	*"Dama" nestii* - The antlers from Casa Frata show a basally inserted basal tine, forming a wide angle with the beam. This morphology matches that of *"eurygonos"* or *'P.' farnetensis*
	Upper Valdarno (upper part: Il Tasso, Le Strette al Tasso, Le Ville, Case Inferno, Colombaiolo, Figline, San Giovanni Valdarno)	Italy	late MNQ 18	Tasso	ca. 1.77	6,8	
	Villa San Faustino	Italy	late MNQ 18	Tasso	ca. 1.8	[43]	
	Monte Riccio	Italy	late MNQ 18	Tasso	ca. 1.8	[44]	*Praeelaphus lyra*[45]
	Dmanisi	Georgia	late MNQ 18	Tasso	ca. 1.8	[46]	*Cervus abesalomi*[47], *Cervus nestii*[12,22,23]
cf.	Carsoli	Italy	MNQ 18	Tasso	ca. 1.8	[48]	
'Pseudodama' farnetensis							
	Val di Chiana	Italy	early MNQ 19	Farneta	ca. 1.5	6	The type of *P. farnetensis* Azzaroli, 1992 (IGF 194V from Le Ville, Farneta) is peculiar because the right antler has four points and the left antler has three points (middle tine is missing). In general morphology, it is quite similar to the type of *"Dama nestii eurygonos"* (IGF 245 from Upper Valdarno).
	Selvella	Italy	early MNQ 19	Farneta	ca. 1.5	6,[49]	
	Pietrafitta	Italy	early MNQ 19	Farneta	ca. 1.5	[50]	*Axis eurygonos*
	Mugello	Italy	early MNQ 19	Farneta	ca. 1.5	[7]	*Axis eurygonos*
	Venta Micena	Spain	early MNQ 19	Farneta	ca. 1.6	[20,51]	*Metacervoceros rhenanus*
	Barranco León 5	Spain	late MNQ 19	Pirro	ca. 1.4	[20,51]	*Metacervoceros rhenanus*
	Fuente Nueva 3	Spain	late MNQ 19	Pirro	ca. 1.3	[20,51]	*Metacervoceros rhenanus*

	Pirro Nord	Italy	late MNQ 19	Pirro	ca. 1.6-1.3	[52]	*Axis eurygonos*
	Capena	Italy	late MNQ 19	Pirro	ca. 1.6-1.3 (?)	[53]	*Dama nestii eurygonos*
?	Zabia cave	Poland	MNQ 18-19	Tasso-Farneta	ca. 1.7-1.3 (?)	[54]	*Dama vallonnetensis* - The specimens are not described; the age of the site fits with an attribution to *P. farnetensis*
'Pseudodama' perolensis							
	Peyrolles	France	early MNQ 19	Farneta	ca. 1.47	6,9,[55]	Debated taxonomy - "The name *Cervus rhenanus* Dubois, 1904 is to be preserved and used; *Cervus philisi* Schaub, 1941, *Cervus perolensis* Bout & Azzaroli, 1952, *Cervus ischnoceros* Boeuf et al., 1992, and *Pseudodama lyra* Azzaroli, 1992, all are junior synonyms. *Cervus pardinensis* Croizet & Jobert, 1828 is a distinct species, it differs from *C. rhenanus* by having cingula on the upper molars"[34]
?	Durfort	France	MNQ 20-21		ca. 1.0-0.5	[56]	"The skull and antlers of a small cervid, referred by Heintz and Poplin (1974) to *"C." elaphoides*, has been examined at MNHN in the present study. Each antler possesses a single lower tine based a few centimetres above the rose, above which the beam, long and sinuous, proceeds without further branching until 28 cm above the rose, at which point it is broken off. This morphology is quite unlike the Mosbach type material of *"C." elaphoides*, and also unlike *C. elaphus* or *D. dama*. Its greatest similarity is perhaps to *Cervus perolensis*, but the true specific attribution of this specimen remains to be established"[52]
'Pseudodama' vallonnetensis							
	Le Vallonnet	France	MNQ 20	Colle Curti	ca. 1.2-1.1	[57,58]	*Cervus* (s.l.) *nestii vallonnetensis*[57], *Pseudodama nestii vallonnetensis*[58]
	Untermassfeld	Germany	MNQ 20	Colle Curti	ca. 1.05	28	*Cervus* s.l. *nestii vallonnetensis*[47,59]
	Collecurti	Italy	MNQ 20	Colle Curti	ca. 1.0	[60]	*Dama nestii*[60], *"Cervus" nestii vallonnetensis*[61], *Pseudodama* ex gr. *nestii*[62]
	Ellera	Italy	MNQ 20	Colle Curti	ca. 1.0	[63]	Under study
?	Monte Peglia	Italy	MNQ 20	Colle Curti	ca. 1.0	[64]	No cranium/antler remains - *Axis eurygonos*[64]. Probably *Pseudodama* sp.
	Frantoio (Arda River)	Italy	MNQ 20	Colle Curti	ca. 1.0	[65]	*Pseudodama farnetensis*
	Cueva Victoria	Spain	MNQ 20	Colle Curti	ca. 1.0	[20,66]	*Dama vallonnetensis*[20], *Dama* cf. *vallonnetensis*[66]
cf.	El Chaparral	Spain	MNQ 20	Colle Curti	ca. 1.0	[67]	*Dama* cf. *vallonnetensis*

	Sainzelles	France	MNQ 20			[22]	*"Dama" vallonnetensis* according to Palombo[22], but without descriptions or references - "These remains are *Dama*-sized, but their morphology does not correspond well to either *D. dama* or *C. elaphus*. For example, the M3's lack the lingual 'step' between second and third lobes characteristic of *D. dama*, and unlike both species [...], possess small lingual cuspules on the anterior part of the third lobe. Their specific attribution is therefore uncertain"[52]
	St. Prest	France	MNQ 20			[52,68]	Dubitative attribution - "The specific identity of the St. Prest material must […] be regarded as unknown, although the rather high position and acute angle of the first tine on SPR-68 makes its attribution to *Dama dama* unlikely"[52]
	Vallparadís Section (Early Pleistocene layers)	Spain	MNQ 20-21	Colle Curti-Slivia	ca. 1.2-0.8	[69]	*Dama vallonnetensis*
	Atapuerca Complex (Sima del Elefante TE8-9-10-14, Gran Dolina TD3-4-5-6-7-8, Penal)	Spain	MNQ 20-21	Colle Curti-Slivia	ca. 1.2-0.7	10,[70–73]	No cranium/antler remains - *Dama nestii? vallonetensis*[70], *Dama vallonnetensis*[71–73]
	Redicicoli1	Italy	MNQ 21	Slivia	ca. 0.8	[7]	*Axis eurygonos*
?	Slivia	Italy	MNQ 21	Slivia	ca. 0.8	[74]	*Dama* sp.

Supplementary Table S2. List of remains of *'Pseudodama' nestii* from Pantalla (Italy). Stratigraphic information is from the excavation notes and from Gentili et al.[34]. L, left; R, right. *Based on the similar fossilization features (e.g., colour) and the occlusion between the upper and lower right teeth, we can hypothesize that these remains belonged to the same male skull. **3D models available at www.morphosource.org (see Methods).

SABAP_UMB	Element	Side	Layer	Figure	Notes
167339	Hemimandible with dp2–m1 and erupting m2	L	Silty sand	S1	
167340	m3 fragment	L	Silty sand	-	
167341	m2	L	Silty sand	-	Covered by sediment
167343	M1–M3	L	Clay (paleosol)	-	
167344	P2	L	Silty sand	1	Associated to cranium 337643
167353	Partial articulated hindlimb with distal tibia, astragalus, calcaneum, lateral malleolus, cubo-navicular, cuneiforms, metatarsal, medial and lateral phalanges I, lateral phalanx II, medial sesamoids	R	Clay (paleosol)	S2	Found in anatomical articulation
167356	Hemimandible fragment with dp3–dp4	L	Clay (paleosol)	S1	
167357	Phalanx II	-	Clay (paleosol)	-	Same individual as 167353
167362	Fragmented phalanx I	-	Clay (paleosol)	-	Middle part missing
337625	Pedicle with basal fragment of antler	L	Silty sand	1**	
337629	Hemimandible with p2–m3	R	Silty sand	S1	
337630*	Hemimandible with p2–m3	R	Silty sand	S1	
337631	Hemimandible with complete dentition	L	Silty sand	S1**	Rostral fragment of right hemimandible with i1 and roots of i2–c1
337632	Mandible with dp2–m1 and erupting m2	L+R	Silty sand	S1	
337633*	Maxillary fragment with P2–M3	R	Silty sand	1	Same individual as 337637?
337637*	Maxillary fragment with P3–M1	L	Silty sand	S1	Same individual as 337633?
337643	Cranium without antlers	-	Silty sand	1**	
337649	Axis, cervical vertebrae III–IV and 3 articulated caudal vertebrae	-	Silty sand	S1	
337651	Metatarsal	R	Silty sand	S2	
337652	Fragmented metacarpal	R	Silty sand	S2	Distal part missing
337655*	Cranium with antlers	-	Silty sand	1	
no num.	M3	R	Clay (paleosol)	-	

Supplementary Table S3. Measurements (mm) of the skeletal material of *'Pseudodama' nestii* from Pantalla (Italy). Measurements after Von den Driesch[75] (see numbers and abbreviations in brackets) and Breda et al.[26] (for antlers). The measurements of the accessory tine of SABAP_UMB 337655 are indicated by +. * The beam base is deformed by the presence of the accessory tine. Estimated measurements are in italics.

Cranium	SABAP_UMB 337625	SABAP_UMB 337633	SABAP_UMB 337643	SABAP_UMB 337655
Short skull length: Basion-Premolare (4)			170.6	
Basicranial axis: Basion-Synsphenion (6)			39.8	36.5
Median frontal length (10)			142.5	
Lambda-Nasion (11)			116.0	
Length of the cheek tooth row (measured along the alveoli) (20)		80.4	77.5	
Length of the molar row (measured along the alveoli on the buccal side) (21)		45.0	44.5	
Length of the premolar row (measured along the alveoli on the buccal side) (22)		37.7	33.4	
Greatest inner length of the orbit: Ectorbitale-Entorbitale (23)			39.0	38.6
Greatest inner height of the orbit (24)			40.4	
Greatest mastoid breadth: Otion-Otion (25)			87.5	98.6
Greatest breadth of the occipital condyles (26)			50.4	54.6
Greatest breadth at the bases of the paraoccipital processes (27)			65.1	82.8
Greatest breadth of the foramen magnum (28)			21.6	23.6
Greatest height of the foramen magnum: Basion-Opisthion (29)			20.4	21.5
Greatest neurocranium breadth: Euryon-Euryon (30)			72.2	81.1
Least frontal breadth: least breadth of the forehead aboral to the orbits (31)			95.5	96.8
Greatest breadth across the orbits: Ectorbitale-Ectorbitale (32)			*110*	126.5
Least breadth between the orbits: Entorbitale-Entorbitale (33)			75.0	
Greatest breadth across the nasals (35)			*40*	

Greatest palatal breadth: measured across the outer borders of the alveoli (37)		*74*	
Basion-highest point of the superior nuchal crest (38)		61.8	62.1
Pedicle length (shortest distance between frontoparietal suture and lower edge of burr)	38.3	28.3 (L) - 26.5 (R)	27.4 (L) - 25.6 (R)
Circumference at the top of the pedicle	9.5	10.6 (L) - 11.0 (R)	9.9 (L) - 9.7 (R)
Anteroposterior diameter at the top of the pedicle (DAP)	28.0	28.4 (L) - 29.1 (R)	28.7 (L) - 29.1 (R)
Mediolateral diameter at the top of the pedicle (DML)	31.3	36.0 (L) - 37.4 (R)	30.5 (L) - 31.3 (R)

**Antlers**	**SABAP_UMB 337625**	**SABAP_UMB 337655**		
	L	L	R	+
Anteroposterior diameter of the base just above the burr	38.0	34.5	36.6	
Mediolateral diameter of the beam just above the burr	36.2	32.1	48.5*	
Basal tine, basal circumference	11.1	8.5	8.0	8.9
Basal tine, anteroposterior basal diameter	32.7	28.6	24.8	24.7
Basal tine, mediolateral basal diameter	24.2	21.5	23.8	25.3
Beam circumference just above basal tine	10.5	9.9	9.5	
Antero-posterior (maximum) diameter of the beam just above basal tine	34.8	27.5	31.8	
Mediolateral (minimum) diameter of the beam just above basal tine	31.6	26.8	27.6	
Basal tine distance from the burr (measured on the anterior side, from the point of max bending on the insertion)	30.2	27.5	36.3	
Basal tine length (from the axilla, along the curvature on the side internal to the bifurcation)	150	143	170	
Angle between pedicle and beam above the basal tine (measured in lateral view)	121°	125°	121°	
Angle between basal tine and beam (in mesial view, parallel to ridges and grooves)	44°	45°	48°	

Upper teeth	SABAP_UMB 167343	SABAP_UMB 167344	SABAP_UMB 337633	SABAP_UMB 337637	SABAP_UMB 337643	SABAP_UMB no num.
P2 length		12.5	12.7	12.8	12.6	
P2 breadth		12.3	11.6	12.0	12.6	
P3 length			13.5	13.2	11.0	
P3 breadth			13.0	14.6	13.7	
P4 length			10.5	11.0	9.0	
P4 breadth			15.0	16.1	15.4	
M1 length	15.3		14.3		13.4	
M1 breadth	16.6		16.5		16.7	
M2 length	18.5		16.3		15.7	
M2 breadth	20.5		18.3		18.4	
M3 length	19.5		17.0		15.3	*18*
M3 breadth	19.0		17.1		18.2	20.1

Mandible	SABAP_UMB 167339	SABAP_UMB 337629	SABAP_UMB 337630	SABAP_UMB 337631	SABAP_UMB 337632	
	L	R	R	L	R	L
Length from the angle: Gonion caudale-Infradentale (1)				217.2		
Length from the condyle: aboral border of the condyle process-Infradentale (2)				*216*		
Length: Gonion caudale-aboral border of the alveolus of m3 (3)				61.1		
Length of the horizontal ramus: aboral border of the alveolus of m3-Infradentale (4)				*158.5*		
Length: Gonion caudale-oral border of the alveolus of p2 (5)				142.5		
Length of the cheek tooth row, measured along the alveoli on the labial side (6)	57.0	87.3	93.9	85.4	59.0	59.0
Length of the molar row, measured along the alveoli on the labial side (7)	15.5	51.5	55.6	51.1	17.1	16.6

Length of the premolar row, measured along the alveoli on the labial side (8)	40.5	35.5	37.7	33.6	40.8	41.4
Length of the diastema, oral border of the alveolus of p2-aboral border of the alveolus of c1 (11)	*48*	*52.5*		67.5		
Aboral height of the vertical ramus: Gonion ventral-highest point of the condyle process (12)				61.2		
Middle height of the vertical ramus: Gonion ventral-deepest point of the mandibular notch (13)				*65.5*		
Height of the mandible: behind m3, from the most aboral point of the alveolus on the labial side (15a)		34.9	35.6			
Height of the mandible: in front of m1 (15b)	24.7	27.4	30.2		24.8	*27*
Height of the mandible: in front of p2 (15c)	19.5	25.3	22.2		20.0	18.0

**Lower teeth**	**SABAP_UMB 167339**	**SABAP_UMB 167356**	**SABAP_UMB 167340**	**SABAP_UMB 167341**	**SABAP_UMB 337629**	**SABAP_UMB 337630**	**SABAP_UMB 337631**	**SABAP_UMB 337632**	
	L	L	L	L	R	R	L	R	L
i1 length							8.8		
i1 breadth							6.2		
i2 length							4.1		
i2 breadth							4.8		
i3 length							3.8		
i3 breadth							4.2		
c1 length							3.0		
c1 breadth							6.7		
dp2 length	9.5							9.3	9.4
dp2 breadth	5.0							5.4	5.1
dp3 length	12.4	13.8						12.7	12.6

dp3 breadth	6.5	6.0						7.0	
dp4 length	18.4	20.3						19.5	19.4
dp4 breadth	9.6	8.2						9.4	
p2 length					10.9	9.5	9.8		
p2 breadth					5.6	5.8	6.5		
p3 length					10.8	13.6	10.2		
p3 breadth					7.2	8.9	8.0		
p4 length					12.3	14.3	12.7		
p4 breadth					8.1	10.1	8.2		
m1 length	15.7				12.8	13.3	12.0	16.7	17.0
m1 breadth	11.1				11.3	11.8	10.9	10.7	
m2 length				17.1	15.1	17.1	15.4		
m2 breadth				12.3	12.5	13.2	12.1		
m3 length					23.0	24.6	23.3		
m3 breadth			11.5		11.8	12.8	11.5		

	**SABAP_UMB 337651**	**SABAP_UMB 337652**
	**Metatarsal**	**Metacarpal**
Greatest length (GL)	219.8	
Greatest breadth of the proximal end (Bp)	25.3	33.5
Greatest depth of the proximal end (Dp)	24.5	24.3
Greatest breadth of the distal end (Bd)	28.7	

Greatest depth of the distal end (Dd)	19.4		
Smallest breadth of the diaphysis (SD)	16.5	22.6	
Smallest depth of the diaphysis (DD)	17.3	19.4	
**SABAP_UMB 167353**			
**Tibia**			
Smallest breadth of the diaphysis (SD)			26.0
Greatest breadth of the distal end (Bd)			39.9
Greatest depth of the distal end (Dd)			29.9
**Astragalus**			
Greatest length on the lateral side (GLl)			44.4
Greatest length on the medial side (GLm)			40.8
Greatest breadth of the distal end (Bd)			27.4
Greatest depth of the lateral half (Dl)			21.3
Greatest depth of the medial half (Dm)			24.4
**Calcaneum**			
Greatest breadth (GB)			31.2
**Cubo-navicular**			
Greatest breadth (GB)			35.5
**Lateral malleolus**			
Greatest depth (GD)			22.4
**Metatarsal**			
Greatest length (GL)			252.0
Greatest breadth of the proximal end (Bp)			31.0
Greatest depth of the proximal end (Dp)			32.5

Greatest breadth of the distal end (Bd)	33.1
Greatest depth of the distal end (Dd)	23.1
Smallest breadth of the diaphysis (SD)	19.7
Smallest depth of the diaphysis (DD)	18.0
**Medial phalanx I**	
Greatest length of the peripheral half (GLpe)	51.3
Greatest breadth of the proximal end (Bp)	17.2
Smallest breadth of the diaphysis (SD)	13.4
Greatest breadth of the distal end (Bd)	15.3
**Lateral phalanx I**	
Greatest length of the peripheral half (GLpe)	53.2
Greatest breadth of the proximal end (Bp)	17.1
Smallest breadth of the diaphysis (SD)	12.8
Greatest breadth of the distal end (Bd)	14.5
**Medial phalanx II**	
Greatest length of the peripheral half (GLpe)	36.2
Greatest breadth of the proximal end (Bp)	15.8
Smallest breadth of the diaphysis (SD)	12.3
Greatest breadth of the distal end (Bd)	12.6
**Lateral phalanx II (SABAP_UMB 167357)**	
Greatest length of the peripheral half (GLpe)	38.0
Greatest breadth of the proximal end (Bp)	16.2
Smallest breadth of the diaphysis (SD)	12.3
Greatest breadth of the distal end (Bd)	12.6

Supplementary Table S4. Age estimations of six individuals from Pantalla based on tooth eruption and wear patterns in lower teeth, following the method developed for the extant fallow deer[76]. No age estimation methods are available based on upper teeth. TWS, tooth wear stage; MWS, mandible wear stage.

	**TWS**					**MWS**	**Age (months)**
	**dp4**	**p4**	**m1**	**m2**	**m3**		
SABAP_UMB 167339	e		c	a		D	5–12
SABAP_UMB 167356	b		?			A-B	< 2
SABAP_UMB 337629			l	j	j	J	118–189
SABAP_UMB 337630		h	i	g	f	I-J	61–189
SABAP_UMB 337631		j	l	i	k	-	> 189
SABAP_UMB 337632	d–e		b	a			5–12

Supplementary Table S5. Measurements of the brain of extinct (*'Pseudodama' nestii* and *Eucladoceros dicranios*) and extant (*Dama dama* and *Cervus elaphus*) deer. *Data from Beccari[36] and Azzaroli[8]. All other measurements are original. BV, brain volume; TeL, length of the telencephalon; TeB, breadth of the telencephalon.

**Species**	**ID**	**Locality**	**Sex**	**BV (mm^3)**	**TeL (mm)**	**TeB (mm)**	**TeB/L %**
'Pseudodama' nestii	SABAP_UMB 337643	Pantalla	Male	197,408.86	94.42	66.24	70
'Pseudodama' nestii	SABAP_UMB 337655	Pantalla	Male	200,667.41	94.17	74.72	79
'Pseudodama' nestii	IGF 1422	Olivola	-	-	ca. 100–110*	ca. 80*	80–72
Eucladoceros dicranios	IGF 2175	Castelfiorentino	-	-	119*	98*	82
Dama dama	CZUFLD FaD001	Western Bohemia	Male	258,964.94	97.93	74.73	76
Dama dama	CZUFLD FaD002	Western Bohemia	Male	226,033.66	96.13	72.26	75
Dama dama	CZUFLD FaD003	Western Bohemia	Female	228,506.55	92.55	71.42	77
Cervus elaphus	CZUFLD ReD001	Western Bohemia	Male	343,561.22	102.65	84.23	82
Cervus elaphus	CZUFLD ReD002	Western Bohemia	Male	353,098.89	104.27	83.37	80
Cervus elaphus	CZUFLD ReD003	Western Bohemia	Female	307,260.02	98.91	83.21	84

Supplementary Table S6. Morphological characters by Lister[39] observed in the teeth of *'Pseudodama' nestii* from Pantalla (Italy). The column on the right indicates whether the character scores as extant *Dama dama* or *Cervus elaphus*. Missing characters could not be scored due to incompleteness and/or other preservation biases of the fossils. Question marks indicate dubitative scoring.

**Upper premolars**	SABAP_UMB 337633, SABAP_UMB 337643, SABAP_UMB 337637
Character 1	*Dama dama*
Character 2	*Dama dama*
**Upper molars**	SABAP_UMB 337633, SABAP_UMB 337643, SABAP_UMB 167343
Character 1	intermediate
Character 3	Intermediate - *C. elaphus*
Character 4	*Cervus elaphus*
Character 5	*Cervus elaphus*
**Lower premolars**	SABAP_UMB 337630
Character 2	*Dama dama*
Character 5	*Dama dama* ?
Character 8	*Dama dama*
**Lower molars**	SABAP_UMB 337630, SABAP_UMB 167339, SABAP_UMB 337632
Character 1	*Dama dama*
Character 2	*Dama dama*
Character 3	*Dama dama*
Character 4	intermediate
Character 5	*Dama dama*
Character 6	*Dama dama*
Character 9	*Dama dama*
Character 11	*Cervus elaphus*

Supplementary Table S7. Morphological characters by Lister[39] observed in the postcranial bones of *'Pseudodama' nestii* from Pantalla (Italy). The column on the right indicates whether the character scores as extant *Dama dama* or *Cervus elaphus*. Missing characters could not be scored due to incompleteness and/or other preservation biases of the fossils. Question marks indicate dubitative scoring.

**Axis**	SABAP_UMB 337649
Character 4	*Dama dama*
**Metacarpal**	SABAP_UMB 337652
Character 1	*Dama dama*
Character 2	*Dama dama* ?
Character 4	*Dama dama*
Character 5	*Dama dama*
**Tibia**	SABAP_UMB 167353
Character 3	*Dama dama*
Character 4	*Dama dama*
Character 5	*Dama dama*
**Astragalus**	SABAP_UMB 167353
Character 1	*Dama dama*
Character 2	*Dama dama*
Character 3	*Dama dama*
Character 4	*Dama dama*
Character 5	*Cervus elaphus*
Character 6	*Cervus elaphus* ?
**Calcaneum**	SABAP_UMB 167353
Character 1	*Dama dama*
**Cubo-navicular**	SABAP_UMB 167353
Character 2	*Dama dama*
**Metatarsal**	SABAP_UMB 167353, SABAP_UMB 337651
Character 1	*Dama dama*
Character 2	*Dama dama*
Character 4	*Dama dama*
Character 5	*Dama dama*
Character 6	*Dama dama* ?
**Phalanx I**	SABAP_UMB 167353
Character 1	*Dama dama*
**Phalanx II**	SABAP_UMB 167353, SABAP_UMB 167357
Character 1	*Dama dama* ?

Supplementary References

[1]. Radulescu, C., Samson, P.M., Petculescu, A. & Stiucã, E. Pliocene large mammals of Romania. *Coloquios de Paleontología* **Vol. Ext. 1**, 549–558 (2003).

[2]. Lacombat, F., Abbazzi, L., Ferretti, M.P., Martínez-Navarro, B., Moullé, P.E. *et al.* New data on the Early Villafranchian fauna from Vialette (Haute-Loire, France) based on the collection of the Crozatier Museum (Le Puy-en-Velay, Haute-Loire, France). *Quatern Int* **179**, 64–71 (2008).

[3]. Fejfar, O., Heinrich, W.D. & Heintz, E. Neues aus dem Villafranchium von Hajnáčka bei Filakovo (Slowakei, ČSSR). *Quartärpaläont* **8**, 47–70 (1990).

[4]. Valli, A.M.F. & Lacombat, F. *"Cervus" pardinensis* (Croizet et Jobert 1828) in *Les grands Mammifères fossiles du Velay* (ed. Lacombat, F.), *Annales des Amis du musée Crozatier* **13/14**, 64–65 (2005).

[5]. Abbazzi, L., Ficcarelli, G. & Torre, D., Deer fauna from the Aulla quarry (Val di Magra, Northern Apennines). Biochronological remarks. *Riv Ital Paleontolol S* **101**, 341–348 (1995).

[6]. Bellucci, L., Biddittu, I., Brilli, M., Conti, J., Germani, M. *et al.* First occurrence of the short-faced bear *Agriotherium* (Ursidae, Carnivora) in Italy: biochronological and palaeoenvironmental implications. *Ital J Geosci* **138**, 124–135 (2019).

[7]. Petronio, C., Bellucci, L., Martinetto, E., Pandolfi, L. & Salari, L. Biochronology and palaeoenvironmental changes from the Middle Pliocene to the Late Pleistocene in Central Italy. *Geodiversitas* **33**, 485–517 (2011).

[8]. Spassov, N. Biochronology and zoogeographic affinities of the Villafranchian faunas of Bulgaria and South Europe. *Hist Nat Bulgar* **12**, 89–128 (2000).

[9]. Viret, J. Le loess à bancs durcis de Saint-Vallier (Drôme) et sa faune de mammifères villafranchiens. Avec une analyse granulométrique. *Nouv Arch Mus Hist Nat Lyon* **4**, 1–200 (1954).

[10]. Malez, M. Kvartarni sisavci (Mammalia) iz Velike Pecine na Ravnoj Gori (SR Hrvatska, Jugoslavija). *Radovi* **1**, 33–139 (1986).

[11]. Kostopoulos, D.S. The Plio-Pleistocene artiodactyls from Macedonia, Greece: 2. The fossiliferous locality of Volakas, VOL (Volakas Basin, Drama, NE Greece). *Paleont Evol* **30-31**, 83–92 (1997).

[12]. Kostopoulos, D.S. & Athanassiou, A. In the shadow of bovids: suids, cervids and giraffids from the Plio-Pleistocene of Greece. *Quaternaire hors-série* **2**, 179–190 (2005).

[13]. de Vos, J., van der Made, J., Athanassiou, A., Lyras, G., Sondaar, P.Y. *et al.* Preliminary note on the Late Pliocene fauna from Vatera (Lesvos, Greece). *Ann Géol Pays Hellén* **39**, 37–70 (2002).

[14]. Barbet, P. *Approche taphonomique du site, Pliocène terminal, de Chilhac (Haute-Loire, France) et étude paléontologique des Cervidae* PhD thesis (Muséum national d'Histoire naturelle Paris, 2006).

[15]. Dubar, M., Guérin, C. & Heintz, E. Les nouveaux gisements Villafranchiens du Ravin de Cornillet (Moustiers Sainte-Marie, Alpes de Haute Provence, France) et leur contexte géologique. *Geobios* **11**, 367–381 (1978).
[16]. Heintz, E. & Dubar, M. Place et signification des dépôts villafranchiens de Moustiers-Ségriès et faune de Mammiféres de Cornillet (Alpes de Houte-Provence). *Bull Mus Natl Hist Nat Paris* **4**, 363–397 (1981).
[17]. Bellucci, L. Bona, F., Corrado, P., Magri, D., Mazzini, I. *et al.* Evidence of late Gelasian dispersal of African fauna at Coste San Giacomo (Anagni Basin, central Italy): Early Pleistocene environments and the background of early human occupation in Europe. *Quat Sci Rev* **96**, 72–85 (2014).
[18]. Bellucci, L., Mazzini, I., Scardia, G., Bruni, L., Parenti, F. *et al.* The site of Coste San Giacomo (Early Pleistocene, central Italy): palaeoenvironmental analysis and biochronological overview. *Quat Int* **267**, 30–39 (2012).
[19]. Soto, E. & Morales, J. Grandes mammiferos del yacimiento villafranquiense de Casablanca I, Almenara (Castellón). *Estudios Geol* **41**, 243–249 (1985).
[20]. Madurell-Malapeira, J. Ros-Montoya, S., Espigares, M.P., Alba, D.M. & Aurell-Garrido, J. Villafranchian large mammals from the Iberian Peninsula: paleobiogeography, paleoecology and dispersal events. *J Iber Geol* **40**, 167–178 (2014).
[21]. Agustí, J., Santos-Cubedo, A., Furió, M., De Marfá, R., Blain, H.A. *et al.* The late Neogene-early Quaternary small vertebrate succession from the Almenara-Casablanca karst complex (Castellón, Eastern Spain): Chronologic and paleoclimatic context. *Quatern Int* **243**, 183–191 (2011).
[22]. Palombo, M.R. Deconstructing mammal dispersals and faunal dynamics in SW Europe during the Quaternary. *Quat Sci Rev* **96**, 50–71 (2014).
[23]. Abbazzi, L., Albianelli, A., Ambrosetti, P., Argenti, P., Basilici, G. *et al.* Paleontological and sedimentological records in Pliocene distal alluvial fan deposit at Cava Toppetti (Todi, Central Italy). *B Soc Paleontol Ital* **36**, 5–22 (1997).
[24]. Schaub, S. Die kleine Hirschart aus dem Oberpliocaen von Senèze (Haute-Loire). *Eclogae Geol Helv* **34**, 264–271 (1941).
[25]. Azanza, B. & Menéndez, E. Los ciervos fósiles del neógeno espanol. *Paleont Evol* **23**, 75–82 (1990).
[26]. Lehmann, U. Weitere Fossilfunde aus dem ältesten Pleistozän der Erpfinger Höhle (Schwäbische Alb). Mit einem Beitrag von E. Bleich. *Mitt Geol Staatsinst Hamburg* **26**, 60–99 (1957).
[27]. Clot, A., Chaline, J., Heintz, E., Jammot, D., Mourer-Chauviré, C. *et al.* Montoussé 5 (Hautes-Pyrénées), un nouveau remplissage de fissure à faune de Vertébrés du Pléistocène inférieur. *Geobios* **9**, 511–514 (1976).
[28]. Garrido, G. La asociación de los géneros *Croizetoceros*, *Metacervoceros* y *Eucladoceros* (Cervidae, Artiodactyla, Mammalia) en el yacimiento de Fonelas P-1 (Cuenca de Guadix, Granada). *Cuad Mus Geomin* **10**, 365–396 (2008).

[29]. Arribas, A., Garrido, G., Viseras, C., Soria, J.M., Pla, S. *et al.* A mammalian lost world in Southwest Europe during the Late Pliocene. *PLoS ONE* **4**, 10.1371/journal.pone.0007127 (2009).
[30]. Boeuf, O., Geraads, D. & Guth, C. Cervidés villafranchiens de Blassac-la-Girondie (Haute-Loire, France). *Ann Paléont* **78**, 159–187 (1992).
[31]. Geraads, D. Contribution des Cervides à la chronologie des débuts de l'occupation humaine en Europe Occidentale. *Quaternaire* **3-4**, 167–174 (1990).
[32]. Brugal, J.P., Ambert, P., Bandet, Y., Leroy, S., Roiron, P. *et al.* Mammifères et végétaux du maar Pliocène final de Nogaret (Escandorgue: Hérault, France). *Geobios* **23**, 231–247 (1990).
[33]. Spaan, A. A revision of the deer from Tegelen (province of Limburg, The Netherlands). *Scripta Geol* **98**, 1–85 (1992).
[34]. de Vos, J., Mol, D. & Reumer J.W.F. Early Pleistocene Cervidae (Mammalia, Artiodactyla) from the Oosterschelde (the Netherlands), with a revision of the cervid genus *Eucladoceros* Falconer, 1868. *Deinsea* **2**, 95–121 (1995).
[35]. Lister, A.M. The stratigraphical significance of deer species in the Cromer Forest-bed Formation. *J Quat Sci* **8**, 95–108 (1993).
[36]. Azzaroli, A. The deer of the Weybourne Crag and Forest Bed of Norfolk. *Bull British Mus Nat Hist Geol* **2**, 3–96 (1953).
[37]. Konidaris, G., Tourloukis, V., Kostopoulos, D.S., Thompson, N., Giusti D. *et al.* Two new vertebrate localities from the Early Pleistocene of Mygdonia Basin (Macedonia, Greece): Preliminary results. *C R Palevol* **14**, 353–362 (2015).
[38]. Boulbes, N., Mayda, S., Titov, V.V. & Alçiçek, M.C. Les grands mammifères du Villafranchien supérieur des travertins du Bassin de Denizli (Sud-Ouest Anatolie, Turquie). *L'anthropologie* **118**, 44–73 (2014).
[39]. Abbazzi, L. *Cervus* sp. (Linné 1750) (=*Pseudodama* p.p. sp.) in *Les grands Mammifères fossiles du Velay* (ed. Lacombat, F.), *Annales des Amis du musée Crozatier* **13/14**, 102–103 (2005).
[40]. Girotti, O., Capasso Barbato, L., Esu, D., Gliozzi, E., Kotsakis, T. *et al.* The section of Torre Picchio (Terni, Umbria, Central Italy): A Villafranchian site rich in vertebrates, molluscs, ostracods and plants. *Riv Ital Paleontolol S* **109**, 77–98 (2003).
[41]. Mazza, P., Bertini, A. & Magi, M. The late Pliocene site of Poggio Rosso (Central Italy): Taphonomy and palaeoevironment. *Palaios* **19**, 227–248 (2004).
[42]. De Giuli, C. & Masini, F. The latest Villafranchian faunas of Italy. The Casa Frata local fauna (Upper Valdarno, Tuscany). *Palaeontogr Ital* **74**, 1–9 (1987).
[43]. Sardella, R., Di Stefano, G. & Petronio, C. The Villafranchian mammal faunas from the Tiber River basin (Umbria, Central Italy). *Il Quaternario* **8**, 509–514 (1995).
[44]. Mazzini, I., Paccara, P., Petronio, C. & Sardella, R. Geological evolution and biochronological evidences of the Monte Riccio section (Tarquinia, central Italy). *Riv Ital Paleontolol S* **106**, 247–256 (2000).

[45]. Croitor, R. Lower Pleistocene ruminants from Monte Riccio (Tarquinia, Italy). *Oltenia* **28**, 221–226 (2012).
[46]. Bukhsianidze, M. Dmanisi aritodactyl assemblage. *XIV EAVP Meeting Abstract Book*, 36 (2016).
[47]. Kahlke, H.D. Neufunde von Cerviden-Resten aus dem Unterpleistozän von Untermassfeld in *Das Pleistozän von Untermassfeld bei Meiningen (Thüringen), vol. 2* (ed. Kahlke, R.D.) 461–482 (Römisch-Germanisches Zentralmuseum, 2001).
[48]. Segre Naldini, E. & Valli, A.M.F. Villafranchian cervids from central Italy. *Quaternaria Nova* **7**, 159–204 (2004).
[49]. De Giuli, C. Late Villafranchian faunas of Italy: The Selvella Local Fauna in the southern Chiana Valley - Umbria. *Palaeontogr Ital* **74**, 11–50 (1986).
[50]. Martinetto, E., Bertini, A., Basilici, G., Baldanza, A., Bizzarri, R. *et al.* The plant record of the Dunarobba and Pietrafitta sites in the Plio-Pleistocene palaeoenvironmental context of Central Italy. *Alpine Med Quat* **27**, 29–72 (2014).
[51]. Abbazzi, L. La fauna de cérvidos de Barranco León y Fuente Nueva 3 in *Ocupaciones Humanas en el Pleistoceno inferior y medio de la Cuenca de Guadix-Baza* (eds. Toro, I., Martínez-Navarro, B. & Agustí, J.) 273–290 (Arqueología Monografías, 2010).
[52]. Petronio, C., Bellucci, L. & Di Stefano, G. *Axis eurygonos* from Pirro Nord (Apricena, Southern Italy). *Palaeontogr Abt A* **298**, 169–181 (2013).
[53]. Petronio, C. *Dama nestii eurygonos* Azz. di Capena (Roma). *Geol Romana* **18**: 105–125 (1979).
[54]. Nadachowski, A., Stefaniak, K., Szynkiewicz, A., Marciszak, A. Socha, P. *et al.* Biostratigraphic importance of the Early Pleistocene fauna from Zabia Cave (Poland) in Central Europe. *Quatern Int* **243**, 204–218 (2011).
[55]. Valli, A.M.F., Caron, J.B., Debard, E., Guérin, C., Pastre, J.F. *et al.* Le gisement paléontologique villafranchien terminal de Peyrolles (Issoire, Puy-de-Dôme, France): Résultats de nouvelles prospections. *Geodiversitas* **28**, 297–317 (2006).
[56]. Lister, A.M. Critical reappraisal of the Middle Pleistocene deer species "*Cervus" elaphoides* Kahlke. *Quaternaire* **3-4**, 175–192 (1990).
[57]. de Lumley, H., Kahlke, H.D., Moigne, A.M. & Moullé, P.É. Les faunes de grands mammifères de la grotte du Vallonnet. *L'anthropologie* **92**, 465–495 (1988).
[58]. Moullé, P.É., Lacombat, F. & Echassoux, A. Apport des grands mammifères de la grotte du Vallonnet (Roquebrune-Cap-Martin, Alpes-Maritimes, France) à la connaissance du cadre biochronologique de la seconde moitié du Pléistocène inférieur d'Europe. *L'anthropologie* **110**, 837–849 (2006).
[59]. Kahlke, H.D. Die Cerviden-Reste aus dem Unterpleistozän von Untermassfeld in *Das Pleistozän von Untermassfeld bei Meiningen (Thüringen), vol. 1* (ed. Kahlke, R.D.) 181–275 (Römisch-Germanisches Zentralmuseum, 1997).
[60]. Ficcarelli, G. & Mazza, P. New fossil findings from the Colfiorito basin (Umbria-Marchean Apennine). *B Soc Paleontol Ital* **29**, 245–247 (1990).

[61]. Ficcarelli, G. & Silvestrini, M. Biochronologic remarks on the Local Fauna of Colle Curti (Colfiorito basin, Umbria-Marchean Apennine, Central Italy). *B Soc Paleontol Ital* **30**, 197–200 (1991).
[62]. Mazza, P.P.A. & Ventra, D. Pleistocene debris-flow deposition of the hippopotamus-bearing Collecurti bonebed (Macerata, Central Italy): Taphonomic and paleoenvironmental analysis. *Palaeogeogr Palaeoclimatol Palaeoecol* **310**, 296–314 (2011).
[63]. Cherin, M., Bizzarri, R., Buratti, N., Caponi, T., Grossi, F. *et al.* Multidisciplinary study of a new Quaternary mammal-bearing site from Ellera di Corciano (central Umbria, Italy): Preliminary data. *Rend Online Soc Geol Ital* **21**, 1075–1077 (2012).
[64]. Petronio, C., Angelone, C., Atzori, P., Famiani, F., Kotsakis, T. *et al.* Review and new data of the fossil remains from Monte Peglia (late Early Pleistocene, central Italy). *Riv Ital Paleontolol S* **126**, 791–819 (2020).
[65]. Bona, F. & Sala, B. Villafranchian-Galerian mammal faunas transition in South-western Europe. The case of the late early Pleistocene mammal fauna of the Frantoio locality, Arda River (Castell'Arquato, Piacenza, northern Italy). *Geobios* **49**, 329–347 (2016).
[66]. Van der Made, J. The latest Early Pleistocene giant deer *Megaloceros novocarthaginiensis* n. sp. and the fallow deer *Dama* cf. *vallonnetensis* from Cueva Victoria (Murcia, Spain). *Mastia* **11-12-13**, 269–323 (2015).
[67]. Pacheco, F.G., Santiago, A., Gutiérrez, J.M., López-García, J.M., Blain, H.A. *et al.* The Early Pleistocene paleontological site in the Sierra del Chaparral (Villaluenga del Rosario, Cádiz, Southwestern Spain). *Quat Int* **24**, 92–104 (2011).
[68]. Guérin, C., Dewolf, Y. & Lautridou, J.P. Révision d'un site paléontologique célèbre: Saint-Prest (Chartres, France). *Geobios* **36**, 55–82 (2003).
[69]. Madurell-Malapeira, J., Minwer-Barakat, R., Alba, D.M., Garcés, M., Gómez, M. *et al.* The Vallparadís section (Terrassa, Iberian Peninsula) and the latest Villafranchian faunas of Europe. *Quat Sci Rev* **29**, 2972–2982 (2010).
[70]. Van der Made, J. First description of the large mammals from the locality of Penal, and updated faunal lists for the Atapuerca ungulates – *Equus altidens*, *Bison* and human dispersal into Western Europe. Quat Int 295, 36–47 (2013).
[71]. Rodríguez, J., Burjachs, F., Cuenca-Bescòs, G., García, N., Van der Made, J. *et al.* One million years of cultural evolution in a stable environment at Atapuerca (Burgos, Spain). *Quat Sci Rev* **30**, 1396–1412 (2011).
[72]. Huguet, R., Vallverdú, J., Rodríguez-Álvarez, X.P., Terradillos-Bernal, M., Bargallò, A. *et al.* Level TE9c of Sima del Elefante (Sierra de Atapuerca, Spain): A comprehensive approach. *Quat Int* **433**, 278–295 (2017).
[73]. Van der Made, J., Rosell, J. & Blasco, R. Faunas from Atapuerca at the Early-Middle Pleistocene limit: The ungulates from level TD8 in the context of climatic change. *Quat Int* **433**, 296–346 (2017).

[74]. Ambrosetti, P., Bartolomei, G., De Giuli, C., Ficcarelli, G. & Torre, D. La breccia ossifera di Slivia (Aurisina - Sistiana) nel Carso di Trieste. *B Soc Paleontol Ital* **18**, 207–220 (1979).
[75]. Von den Driesch, A. A guide to the measurement of animal bones from archaeological sites. *Peabody Museum Bull* **1**, 1–137 (1976).
[76]. Bowen, F., Carden, R.F., Daujat, J., Grouard, S., Miller, H. *et al. Dama* dentition: A new tooth eruption and wear method for assessing the age of fallow deer (*Dama dama*). *Int J Osteoarchaeol* **26**, 1089–1098 (2016).
